# Docetaxel induced activation of GSDME pathway and pyroptosis enhance immune lethality in prostate cancer cells

**DOI:** 10.1186/s13046-025-03614-1

**Published:** 2025-12-18

**Authors:** Ruoyang Liu, Long Zhang, Guoqing Xie, Xiang Li, Yu Liu, Ningyang Li, Aravind Raveendran, Yuankang Feng, Fubo Lu, Xiyue Deng, Junyi Li, Jinjian Yang, Zhenlin Huang, Zhankui Jia

**Affiliations:** 1 Department of Urology, the First Affiliated Hospital of Zhengzhou University, Zhengzhou, Henan Province China; 2 Key Laboratory of Urinary Tumors, Henan Provincial Health Commission, Zhengzhou, Henan Province China; 3 Department of Urology, The First Affiliated Hospital of Jinzhou Medical University, Jinzhou, Liaoning China

**Keywords:** Prostate cancer, Pyroptosis, Combination therapy, Immunity, GSDME, SKP2

## Abstract

**Background:**

Immunotherapy has emerged as a promising approach in prostate cancer treatment, albeit with less efficacy compared to lung and esophageal cancers. Recent studies have explored the combination of chemotherapy, specifically docetaxel, with immunotherapy to enhance treatment outcomes. Docetaxel has been reported to be associated with the pyroptosis pathway, which can alter the tumor microenvironment. Therefore, we attempted to explore the specific molecular mechanism by which docetaxel enhances immunotherapy, with the aim of providing new insights for the immunotherapy of prostate cancer patients.

**Methods:**

Immunohistochemical analysis was employed to assess changes in immune markers in prostate cancer tissues pre- and post-docetaxel treatment. Functional assays, including LDH release and flow cytometry, validated cell responses. Molecular interactions were investigated using co-immunoprecipitation and GST pull-down assays to elucidate the binding mechanism between GSDME and SKP2. The detailed mechanism of GSDME activating cellular immunity was analyzed by single cell sequencing. A xenograft model was utilized to confirm the therapeutic efficacy and molecular role of docetaxel combined with immunotherapy.

**Results:**

Docetaxel chemotherapy increased immune-related lymphocytes in patient samples, suggesting an enhanced immune response. Furthermore, docetaxel induced pyroptosis in prostate cancer cells via the GSDME pathway, influencing the immune microenvironment without affecting cell viability. GSDME was found to undergo SKP2-mediated ubiquitination and degradation via the proteasome pathway, this process can be blocked by the inhibition of GSDME phosphorylation through the AKT pathway mediated by docetaxel. The results of single-cell sequencing suggest that GSDME can recruit CD8 + T lymphocytes and NK cells in prostate cancer. The experimental data indicate that the combination of docetaxel and avelumab can achieve better therapeutic effects.

**Conclusion:**

Our findings suggest that docetaxel enhances immunotherapy efficacy in prostate cancer by modulating pyroptosis pathways. This approach may offer a novel therapeutic strategy for clinical management.

**Supplementary Information:**

The online version contains supplementary material available at 10.1186/s13046-025-03614-1.

## Background

Given that prostate cancer is the fifth leading cause of male cancer-related deaths, comprehensive research into its underlying mechanisms of occurrence and progression is of paramount importance [1]. PD-1/PD-L1 inhibitors represent a current focal point in cancer treatment research, including investigations of their application in PCa. However, the efficacy of immunotherapy in PCa remains notably inferior to that observed in lung cancer, esophageal cancer, and other malignancies [2]. Over the past decade, substantial evidence has underscored the close relationship between prostate cancer and the immune system. As our understanding of the immune microenvironment has increased, various combination therapeutic approaches, including PD-1/PD-L1 inhibitors, have made significant strides. This resurgence has reignited strong potential for immunotherapy in the management of prostate cancer [3].

The characteristics of the tumor microenvironment (TME) play a crucial role in determining the efficacy of cancer immunotherapy. However, the fundamental connection between immunity and inflammation, which are the basic constituents of TME, remains unclear [4]. Inflammation triggers a significant infiltration of immune cells, such as macrophages and neutrophils, into the affected areas. Upon activation, these immune cells release various inflammatory factors such as TNF-α, IL-6, and IL-1β These factors not only influence the immune milieu, but also have the potential to directly impact the tumor response to immunotherapy [5, 6].

Pyroptosis, a type of programmed cell death, results in the release of interleukin-1β (IL-1β) and interleukin-18 (IL-18). These cytokines attract additional inflammatory cells, intensify the inflammatory response, and significantly influence the cellular immune microenvironment [7]. In the context of pyroptosis, GSDMD and GSDME play pivotal roles and activities. GSDME, also known as DFNA5 (associated with autosomal dominant non-syndromic deafness), has recently been identified as a critical factor in caspase-3-mediated pyroptosis, particularly in chemotherapy-induced scenarios [8, 9]. Therefore, GSDME may be implicated in alterations of the immune microenvironment induced by chemotherapeutic drugs, such as docetaxel.

In recent years, many studies have shown a close link between docetaxel and the immune microenvironment. This drug can directly kill highly proliferative cells (e.g., T cells), but its effects on lymphocytes are complex due to immune pathway activation, drug dosage/duration differences, body compensatory responses, and individual variations. Clinicians may adjust the regimen when significant myelosuppression (often with neutropenia as the first symptom) is observed, further complicating the situation. And numerous studies seem to report that docetaxel actually has a positive feedback effect on the human immune response during its actual use [10–13]. Given this, an in-depth exploration of the molecular mechanism by which docetaxel enhances the efficacy of immunotherapy will have certain scientific significance and clinical application value.

SKP2 is a well-known component of the SKP2-SCF E3 ligase complex. It can bind both K48-linked and K63-linked ubiquitin chains to various substrates. This binding can lead to proteasome-mediated degradation or regulate the function of the targeted substrate [14]. Most importantly, SKP2 plays a crucial role in numerous cancer-associated signaling pathways, highlighting its potential as a target for tumor-related research [15].

In our study, we observed that the E3 ligase SKP2 facilitates the ubiquitination and degradation of GSDME. Docetaxel inhibits this process by potentially modulating the phosphorylation of GSDME by inhibiting the PI3K‒AKT pathway. This inhibition attenuates the impact of SKP2 on GSDME ubiquitination and degradation.

Consequently, docetaxel preserves the GSDME function, while single-cell sequencing revealed that GSDME can induce immune cell infiltration, thereby influencing the immune microenvironment of prostate tumors and enhancing the efficacy of avelumab. Therefore, combining docetaxel with avelumab enhances anticancer efficacy in prostate cancer.

## Methods

### Cell lines, cell culture, and transfection

The DU145, RM1, and 293 T cell lines were obtained from the Cell Bank of the Chinese Academy of Sciences (Shanghai, China). DU145 and RM1 cells were maintained in RPMI-1640 medium, 293 T cells were cultured in DMEM, and DU145 cells were grown in MEM, with all media supplemented with 10% FBS. The cells were cultured in the logarithmic growth phase. Transfections were carried out using Lipofectamine 2000 (Thermo Fisher Scientific), according to the manufacturer’s instructions.

### Immunohistochemistry

Tissue paraffin sections were first immersed in xylene solution for 10 min and then sequentially treated with 100%, 95%, 80%, and 70% ethanol solutions for 2 min each to achieve dehydration. The sections were then rinsed with distilled water for 5 min. To inhibit endogenous peroxidase activity, samples were treated with methanol and 30% hydrogen peroxide. The samples were then washed three times with PBS for 3 min each and blocked with serum at room temperature for 30 min. The paraffin-embedded tissues were sectioned into 5-μm slices and subjected to immunohistochemical staining to assess the expression of CD3 (1:1000; GB12014; Servicebio), CD4 (1:1000; GB15064; Servicebio), CD8 (1:1000; GB114123; Servicebio and GB114196; Servicebio), CD16 (1:2000; GB113963; Servicebio), CD56 (1:1000; GB12041; Servicebio), and NK1.1 (1:100; PK136; Thermo Fisher). The sections were incubated overnight at 4 °C with diluted primary antibodies and washed thrice with fresh PBS. The samples were subsequently incubated with a biotin-labeled secondary antibody (GB23303; Servicebio, Wuhan, China) for 50 min at room temperature. Positive cells were visualized using diaminobenzidine solution (K5007; DAKO, Santa Clara, CA, USA) and examined under a light microscope (Leica DM2700). Immunohistochemistry results were independently evaluated by two senior pathologists in a blinded manner.

### Reverse transcription‒quantitative polymerase chain reaction (RT‒qPCR)

Total RNA was first treated with genomic DNA (gDNA) Eraser Buffer at 36 °C for 30 min to eliminate any genomic DNA. cDNA was synthesized using a cDNA synthesis kit (Takara Bio Inc., Kusatsu, Japan), according to the manufacturer's protocol. Quantitative PCR (qPCR) was conducted using SYBR Green Mix (Roche Diagnostics, Basel, Switzerland) and a QuantStudio 3 Real-Time PCR System (Thermo Fisher Scientific, Inc.), following the manufacturer's instructions. The qPCRs were carried out in a 20 µL system under the following conditions: an initial step at 95 °C for 30 s, followed by 45 cycles of 95 °C for 10 s, 60 °C for 30 s, and 72 °C for 10 s. The relative expression levels of the target genes were calculated using the 2-ΔΔCt method, with ACTB as the internal control. Primer information is provided in Supplementary Table 1.

### Western blot

Cells were lysed using radioimmunoprecipitation assay (RIPA) buffer (cat. no. R0010; Solarbio), and the protein concentrations were measured using a BCA protein assay kit (Beijing Leagene Biotech Co., Ltd.). Protein samples (25 µg per lane) were separated by SDS‒PAGE on 10% gels and transferred to PVDF membranes.

The membranes were blocked with protein-free rapid blocking buffer (Epizyme Pharmaceutical Biotechnology Co., Ltd.) for 15 min at room temperature and incubated overnight at 4 °C with primary antibodies against GSDME (1:500, sc-393162, Santa Cruz), SKP2 (1:500, sc-74477, Santa Cruz), c-MYC tag (1:1000, 341173, ZENBIO), Flag (1:1000, 201126-3A6, ZENBIO), and GAPDH (1:500, sc-47724, Santa Cruz). After primary antibody incubation, membranes were incubated with either DyLight 800-goat anti-rabbit IgG (1:100, A23910, Abbkine) or DyLight 800-goat anti-mouse IgG secondary antibodies (1:100, A23920, Abbkine) for 1 h at 25 °C. The membranes were then scanned using an imaging system (ODYSSEY® CLx, Gene Company Limited) and the optical density was analyzed using Image Studio Lite (LI-COR Biosciences).

### Coimmunoprecipitation

The cells were lysed on ice for 30 min in 1 mL lysis buffer (Servicebio, G2038) containing the protease inhibitor MG132 (Selleck, S2619). After centrifugation at 17,000 g for 30 min, the supernatant was collected. An aliquot of 100 µL was set aside as the input sample, and the remaining supernatant was incubated overnight at 4 °C with protein A + G agarose IP reagent (Bioworld, BD0048) and either 10 µL of anti-FLAG antibody (ZENBIO, T201126-3A6) or GSDME antibody (1:500, sc-393162, Santa Cruz). The beads were washed three times with lysis buffer and boiled in 2X SDS loading buffer for 5 min to elute the bound proteins. Western blot analysis was performed to detect proteins in the immunoprecipitation (IP) samples.

### Colony formation assay

To assess cell proliferation, colony formation assay was performed. Approximately 3,000 cells were plated in each well of a 6-well plate and 2 mL of MEM supplemented with 10% FBS was used. After seven days of incubation, the cells were fixed with paraformaldehyde for 30 min. The colonies were then stained with crystal violet solution for 2 h. Images of the colonies were captured using a microscope (Leica DM IRB; Wetzlar, Germany).

### CCK8 assay

Cells were seeded in 96-well plates at a density of 3 × 103 cells/well. At specified time points (0, 1, 2, 3, and 4 days post-transfection), the cell medium in each well was replaced with 100 μL of fresh medium containing 10 μL of CCK-8 reagent (Dojindo, Tokyo, Japan). The plates were then incubated for 1 h. Optical density at 450 nm (OD450) was measured using a DNM-9606 microplate reader (Perlong, Beijing, China).

### LDH release assay

The cells were seeded in 96-well plates at a density of 3 × 10^4 cells per well. At the indicated time points, the cell medium was replaced with 200 μL Dye Solution from the CytoTox 96® Non-Radioactive Cytotoxicity Assay. The cells were then incubated for 1 h. Optical density at 450 nm (OD450) was measured using a DNM-9606 microplate reader (Perlong, Beijing, China).

### ELISA

Sample and standard were added to enzyme labeling plate wells, shaken, and incubated at 37 °C for 30 min. After washing, enzyme-labeled reagent was added, followed by another incubation. Color development was achieved with agents A and B, and the reaction terminated with a termination solution. Absorbance was measured at 450 nm to detect IL-1β, IL-6, IL-8, IL-10, IL-15, IL-18, and TNF-α levels using specific ELISA kits. (mouse IL-1β ELISA kit, MEIMIAN MM-0040M1, China); (mouse IL-6 ELISA kit, MEIMIAN, MM-0163M2, China); (mouse IL-8 ELISA kit, MEIMIAN MM-0123M2, China); (mouse IL-10 ELISA kit, MEIMIAN MM-0176M2, China); (mouse IL-15 ELISA kit, MEIMIAN MM-0172M1, China); (mouse IL-18 ELISA kit, MEIMIAN MM-0169M1, China); and (mouse TNF-α ELISA kit, MEIMIAN MM-0132M2, China).

### Molecular docking study

The docking study of GSDME and SKP2 protein interactions was conducted using the ZDOCK module in Discovery Studio 3.0, which employs rigid docking techniques. To investigate the phosphorylation of GSDME at S252, simulations were performed using the Amber18. The most stable structure obtained from these simulations was subjected to additional molecular docking with SKP2.

### In vivo tumor xenograft model

All C57BL/6 mice were obtained from the Sipeifu Company (Beijing, China). Approximately 1 × 106 cells were mixed with Matrigel (Becton, Dickinson and Company, USA) at a 1:1 ratio and drawn into a 1 mL syringe. The needle was inserted into the groin at a 45° angle for the injection. After the injection, the pinhole was gently pressed with the left index finger for approximately 1 min, and the mice were then returned to their cages. After the designated period, the mice were euthanized and the tumor masses were removed and photographed. All animal procedures were approved by the Ethics Committee of the First Affiliated Hospital of Zhengzhou University (2024-KY-0595).

### Single-cell sequence

Uterine cell scRNA-seq library was prepared using BMKMANU protocol. Cells were captured with DG1000 system, and libraries constructed with BMKMANU kit. Sequencing was done on Illumina NovaSeq 6000, and FASTQ files processed with BSCMATRIX to generate matrices. Data were aligned to GRCm38 mouse genome, yielding 15,004 cells from control and ASO groups. Downstream analyses used 'Seurat' R package, with low-quality cells filtered based on criteria: cells with fewer than 200 expressed genes, raw counts below 800, or mitochondrial gene expression exceeding 15%. To identify and exclude potential doublets, the ‘DoubletFinder’ R package was employed [16]. Additionally, environmental RNA contamination was removed via the ‘decontX’ R package [17]. A set of 2,000 highly variable genes underwent PCA. Batch effects were corrected using the 'harmony' R package. The top 30 PCs were used for KNN clustering and UMAP visualization. Cell clusters were annotated with marker genes for cell type characterization.

### Statistical analysis

Statistical analyses were conducted via SPSS 22.0 (IBM, USA) and GraphPad Prism 9.3 (GraphPad Software Inc., USA). Qualitative data were presented as percentages. Quantitative data are expressed as mean ± standard deviation (SD) for normally distributed data or as interquartile range for non-normally distributed data. Group comparisons were performed using t-tests, one-way analysis of variance, or χ^2^ tests as appropriate. Pearson’s correlation analysis was used to assess the relationships between the variables. Single-cell RNA-seq data preprocessing and downstream analyses were performed in R (v4.3.2) and Python (v3.10) using the Seurat and Scanpy workflows. All statistical tests were two-tailed, and a p-value of < 0.05 was considered to indicate statistical significance. All experiments were conducted in triplicate or more.

## Results

### Docetaxel affects immune lymphocytes changes

**Fig. 1:**
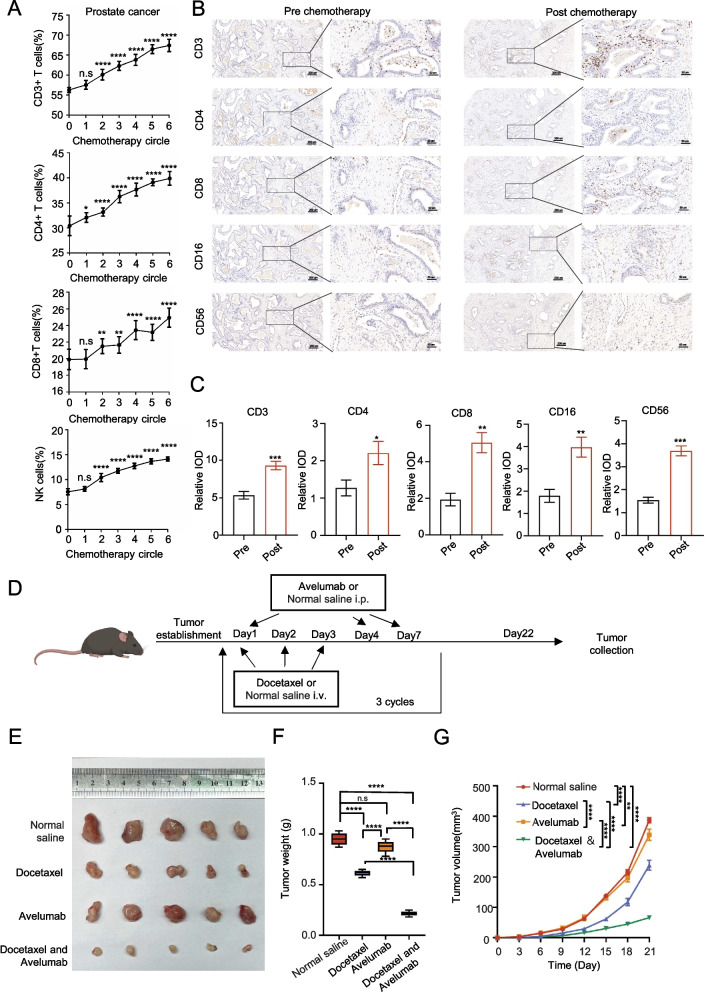
Docetaxel affects immune lymphocytes changes. **A**: Proportion of immune lymphocytes during the prostate cancer chemotherapy cycle. **B**: Immunohistochemistry of immune-related indicators before and after chemotherapy. **C**: IOD values of each immune index before and after chemotherapy. **D**. Schematic diagram of the mouse subcutaneous tumor model. **E**: Tumors from each group were harvested and photographed. **F**: Tumor weight in each group. **G**: Tumor volume at each time point. Data are presented as mean ± SD (*n* = 5). n.s., not significant; *P < 0.01, **P < 0.01, ***P < 0.001

Docetaxel, a widely used chemotherapeutic, has shown promise in combination with immunotherapy for prostate cancer. Our analysis of immune lymphocytes data from prostate cancer patients treated with docetaxel revealed increases in various immune cell subsets (Fig. 1A and Supplementary Fig. 1 A), suggesting its potential to enhance immunity beyond traditionally responsive cancers. Further examination of prostate tumor samples demonstrated enhanced immunohistochemical staining for immune markers after docetaxel treatment (Fig. 1B and C), indicating both systemic and local immune effects. Additionally, GEO database analysis showed docetaxel-induced changes in immune-related gene expression (Supplementary Fig. 1B and C). To assess docetaxel's potential to enhance immunotherapy efficacy in prostate cancer, we established a mouse model and administered treatments (Fig. 1D). Results showed that docetaxel alone was more effective than control, while avelumab alone was less effective. Importantly, the combination of docetaxel and avelumab resulted in the greatest tumor inhibition (Fig. 1E-G).

These findings suggest that docetaxel is a promising candidate for enhancing immunotherapy in prostate cancer.

### Docetaxel induces cell death in prostate cancer cells

**Fig. 2:**
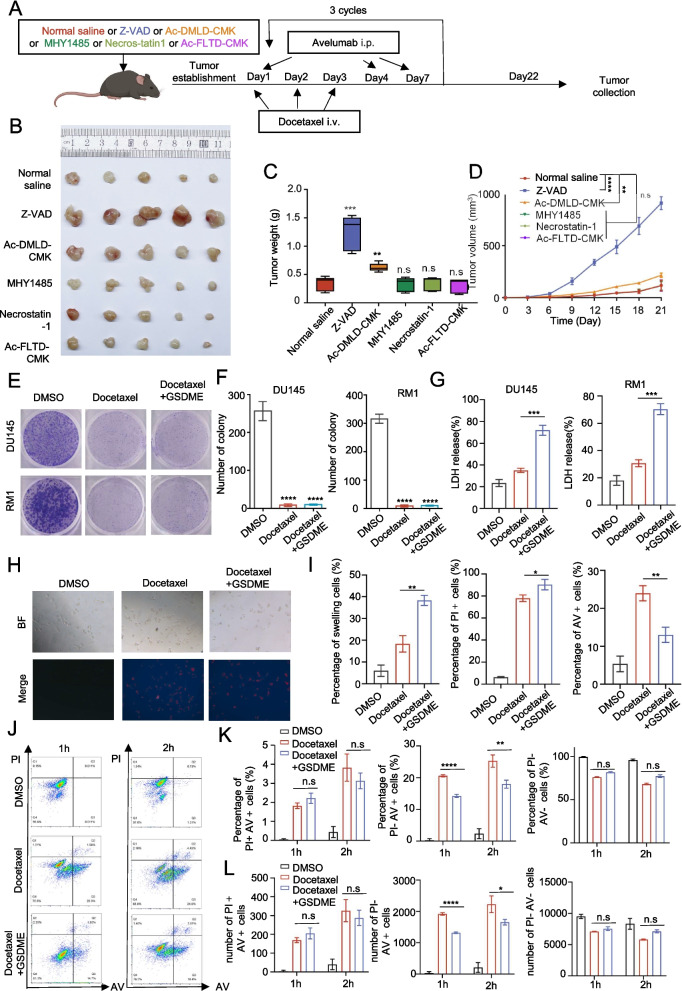
Docetaxel can induce a shift in the mode of death of prostate cancer cells. **A**: Schematic representation of the mouse subcutaneous tumor model. **B**: Tumors from each group were harvested and photographed. **C**: Tumor weight in each group. **D**: Tumor volume at each time point. **E** and **F**: Colony formation assay on colony numbers of the RM1 and DU145 cells. **G**: LDH release assay of LDH release in RM1 and DU145 cells. **H**: Representative images of tumor cells at the indicated time points. PI, propidium iodide; BF, bright field. Results are representative of at least three independent experiments. **I**: Percentage of swollen, AV-positive, and PI-positive cells. **J**: Representative flow cytometry plots of tumor cells undergoing spontaneous cell death. The results are representative of at least three biological replicates. **K**: Percentage of AV − PI −, AV + PI −, and AV + PI + cells at the indicated time points. **L**: Number of AV − PI-, AV + PI-, and AV + PI + cells at the indicated time points. Data are presented as mean ± SD (*n* = 3). n.s., not significant; *P < 0.01, **P < 0.01, ***P < 0.001

It is well known that docetaxel mainly inhibits tumor progression by inhibiting apoptosis [18]. To explore the mechanisms underlying the enhanced immunotherapeutic efficacy of docetaxel, we administered docetaxel in combination with various apoptosis-related inhibitors, including the pan-caspase inhibitor Z-VAD, GSDME inhibitor (Ac-DMLD-CMK), autophagy inhibitor (MYH-1458), RIPK1 inhibitor (necrostatin-1), and GSDMD inhibitor (Ac-FLTD-CMK), in combination with avelumab (Fig. 2A). Analysis of the tumor images, weights, and volumes revealed that the treatment efficacy significantly deteriorated with the use of the broad-spectrum caspase inhibitor Z-VAD, moderately decreased with the GSDME inhibitor, and did not significantly change with the other inhibitors (Fig. 2B, C, and D). Survival analysis confirmed that the combination group had the longest survival, followed by docetaxel alone, while avelumab alone had the worst outcomes (Supplementary Fig. 2 A and B). To further validate the resistance efficacy of the drug when used alone against docetaxel, we conducted in vitro experiments to evaluate two drugs capable of resisting the combined therapeutic effects. The results indicated that Z-VAD could partially counteract the cytotoxic effects of docetaxel. Notably, the Caspase3-GSDME inhibitor Ac-DMLD-CMK failed to resist the cytotoxic effects of docetaxel, suggesting that the GSDME molecule may function in activating immune-related pathways rather than exerting resistance effects on the classical apoptotic pathway of docetaxel (Supplementary Fig. 2 C and D).

These findings suggest that the caspase-3‒GSDME pathway may play a pivotal role in enhancing the effectiveness of the combination therapy.

To further validate the impact of docetaxel on the caspase-3-GSDME pathway, we chose DU145 and RM1 cells for the experiment (Supplementary Fig. 3 A and B). Western blotting after treatment with docetaxel indicated that docetaxel predominantly activated caspase-3 in prostate cancer cells (Supplementary Fig. 3 C). Recent studies have reported that chemotherapeutic drugs induce caspase-3 activation, which in turn triggers pyroptosis by cleaving GSDME [9]. To investigate the role of docetaxel and GSDME in prostate cancer, we conducted experiments focusing on their effects on cellular processes. First, we constructed MYC-GSDME overexpression plasmids and verified their efficacy in DU145 and RM1 cells (Supplementary Fig. 3D and 3E).

We conducted a series of assays to evaluate cell proliferation and pyroptosis in control, docetaxel-treated, and GSDME-overexpressing prostate cancer cells. The colony formation and CCK-8 assays showed significantly inhibited cell proliferation in both docetaxel-treated and GSDME-overexpressing groups compared to control, with GSDME overexpression having minimal effect on total cell death under docetaxel (Fig. 2E-F and Supplementary Fig. 2 F). LDH release was moderately increased by docetaxel, and further increased by GSDME overexpression, while GSDME knockdown decreased LDH release (Fig. 2G and Supplementary Fig. 2G), suggesting increased pyroptosis.

Annexin V staining revealed a decrease in apoptotic cells in the GSDME-overexpressing group compared to wild-type control, with both swollen and shrunken cells showing PI-positive staining indicative of pyroptosis (Fig. 2H-I). Flow cytometry analysis at various time points confirmed these observations, showing a decrease in AV + cells with GSDME overexpression, but no significant change in PI + AV + double-positive cells (Fig. 2J-L).

Collectively, these findings suggest that docetaxel induces pyroptosis in prostate cancer cells, and GSDME overexpression shifts the balance from apoptosis to pyroptosis.

### SKP2 ubiquitinates and modifies GSDME and promotes its degradation

**Fig. 3:**
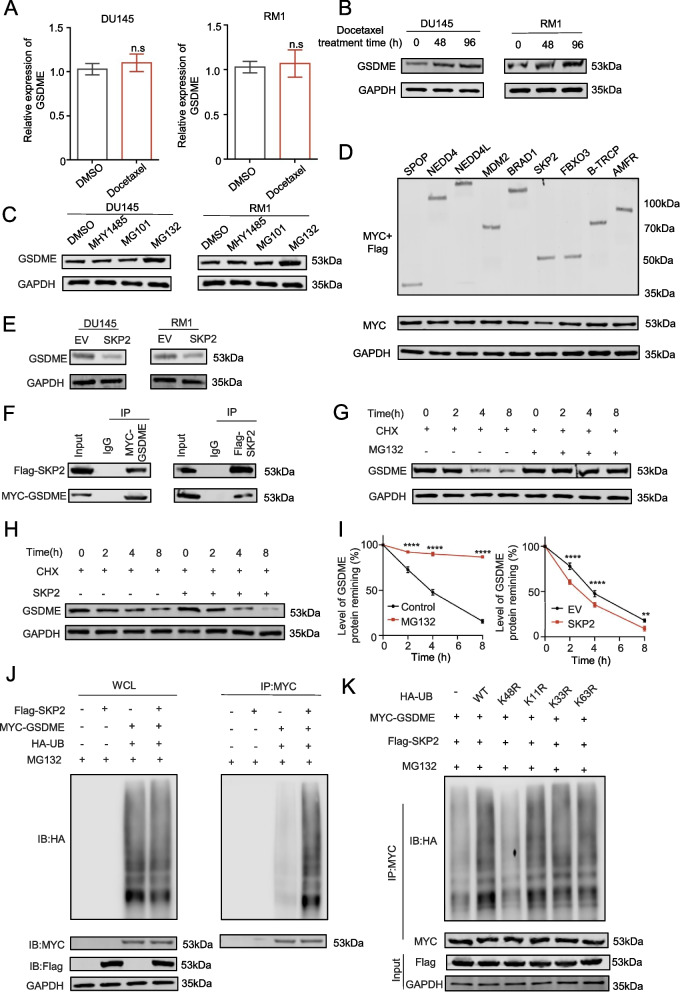
SKP2 ubiquitinates and modifies GSDME and promotes its degradation. **A**: qPCR analysis of GSDME expression before and after docetaxel treatment. **B**: Protein expression of GSDME before and after docetaxel treatment. **C**: Protein expression of GSDME after addition of different drugs. **D**: Protein expression of GSDME after ectopic transfection with different E3s. **E**: GSDME protein expression after SKP2 overexpression. **F**: Ectopia SKP2 and GSDME binding. **G** and **I**: Western blot analysis of GSDME protein expression after MG132 treatment with cycloheximide (CHX) (50 μg/mL). **H** and **I**: Western blot analysis of GSDME protein expression after SKP2 overexpression with cycloheximide (CHX) (50 μg/mL). **J**: Ubiquitination experiment on the changes in GSDME ubiquitination levels after SKP2 was overexpressed with MG132. **K**: Ubiquitination experiment on changes in GSDME ubiquitination levels after ectopic transfection with SKP2 and different lysis mutants of UB. Data are presented as mean ± SD (*n* = 3). n.s., not significant; **P < 0.01, ***P < 0.001, ****P < 0.001

To investigate the mechanisms of docetaxel-GSDME interaction, we examined GSDME expression following docetaxel treatment using PCR and Western blotting. While GSDME mRNA levels remained unchanged (Fig. 3A), protein levels increased with prolonged docetaxel exposure (Fig. 3B). Among various prostate cancer drugs, only docetaxel significantly elevated GSDME expression (Supplementary Fig. 4 A).

Inhibitor studies targeting autophagy, calpain, and proteasomes revealed that MG132 (proteasome inhibitor) increased GSDME expression compared to control and other inhibitors (Fig. 3C), suggesting involvement of the ubiquitin–proteasome pathway. To demonstrate that docetaxel regulates the stability of substrate molecules through the proteasome pathway, we used the proteasome-activating peptide 1 (TFA), which is a ubiquitin proteasome pathway activator and can accelerate the degradation process of ubiquitin-labeled proteins [19]. The results indicated that after intervention with TFA, the expression level of GSDME protein decreased to a certain extent, and it had a certain resistance effect on the expression level of GSDME after docetaxel intervention (Supplementary Fig. 4B). This further suggests that docetaxel may regulate the GSMDE molecule in prostate cancer through the proteasome pathway. Screening identified SKP2 as a candidate E3 ubiquitin ligase that inhibited GSDME protein expression (Fig. 3D), which was confirmed in RM1 and DU145 cells (Fig. 3E).

Co-immunoprecipitation assays confirmed SKP2-GSDME interaction (Fig. 3F). Half-life degradation experiments showed that MG132 inhibited GSDME degradation, an effect exacerbated by SKP2 overexpression (Fig. 3G-I). SKP2 overexpression also increased ubiquitinated GSDME levels (Fig. 3J). Mutational analysis revealed that K11R, K33R, K48R, and K63R ubiquitin mutations promoted SKP2-mediated GSDME ubiquitination, while K48R-ubiquitin inhibited it (Fig. 3K). These findings highlight SKP2 as a key regulator of GSDME ubiquitination and degradation in response to docetaxel.

### The N-terminal amino acid of GSDME binds to SKP2 at the LRR segment and F-box segment

**Fig. 4:**
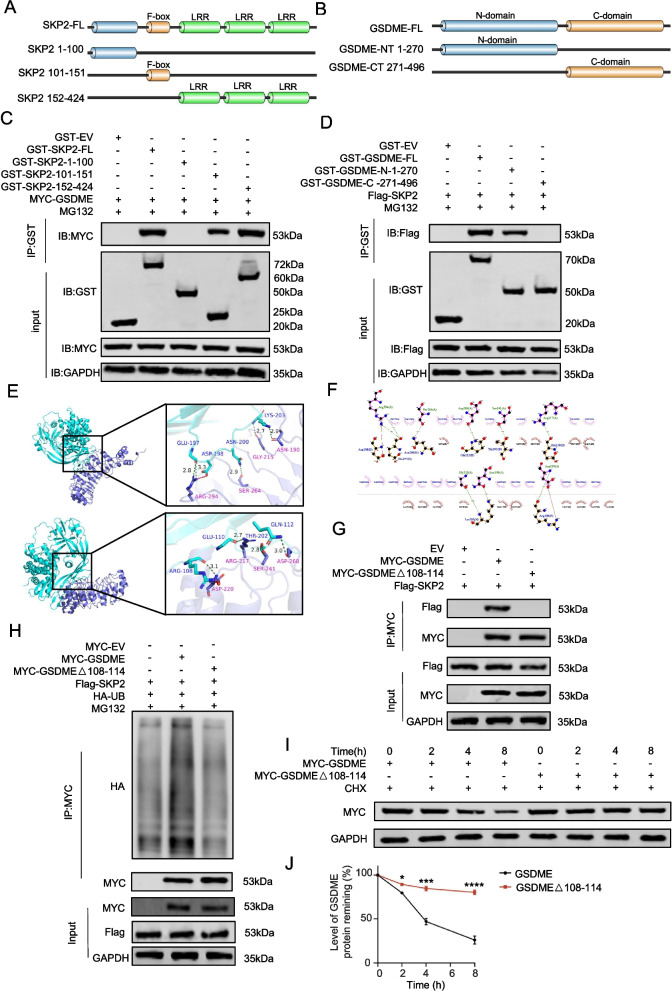
The N-terminal amino acid of GSDME binds to SKP2 at the LRR segment and F-box segment. **A** and **B**: Schematic diagrams depicting a set of recombinant protein constructs of SKP2 and GSDME. **C**: Western blot analysis of GSDME proteins in DU145 whole-cell lysates pulled down by recombinant GST or GST-SKP2 proteins. **D**: Western blot analysis of SKP2 proteins in DU145 whole-cell lysates pulled down by GST or GST-GSDME recombinant proteins. **E** and **F**: Schematic diagrams depicting the potential binding motif of GSDME with SKP2 protein and its molecular docking with GSDME (AlphaFold: AF-O60443-F1) and SKP2 (AlphaFold: AF-Q13309-F1). **G**: Co-IP analysis of the binding of MYC-tagged WT GSDME or mutant GSDME with ectopically expressed FLAG-tagged GSDME treated with MG132. **H**: Western blot analysis of ubiquitination levels in ectopically expressed Myc-tagged WT GSDME or mutant GSDME with ectopically expressed FLAG-tagged SKP2 in 293 T cells treated with MG132. **I** and **J**: Western blot analysis of GSDME protein expression after GSDME or mut-GSDME overexpression with cycloheximide (CHX) (50 μg/mL). Data are presented as mean ± SD (*n* = 3). n.s. not significant; **P < 0.01, ***P < 0.001, ****P < 0.001

To identify binding motifs between GSDME and SKP2, we generated GST-tagged recombinant proteins (Fig. 4A, B). GST pull-down assays showed that SKP2's LRR and F-box domains interact with GSDME (Fig. 4C), while GSDME's N-terminus binds to SKP2 (Fig. 4D). Molecular docking using Z-DOCK confirmed a strong binding affinity (high Z-score) and revealed specific binding sites in GSDME's C-terminus, including ARG-108, GLU-110, GLN-112, GLU197, ASP198, ASN200, THR202, and LYS203 (Fig. 4E, F).

Functional validation was performed using a GSDME deletion mutant lacking the 108–114 region. In prostate cancer cells, this mutant showed reduced binding to SKP2 and decreased SKP2-mediated ubiquitination of GSDME (Fig. 4G, H). A CHX experiment revealed a significant decrease in GSDME degradation rate after mutation (Fig. 4I, J). These findings provide critical insights into SKP2-mediated GSDME ubiquitination and degradation, suggesting potential therapeutic targets for prostate cancer.

### Phosphorylation at the S252 site leads to the degradation of GSDME

**Fig. 5:**
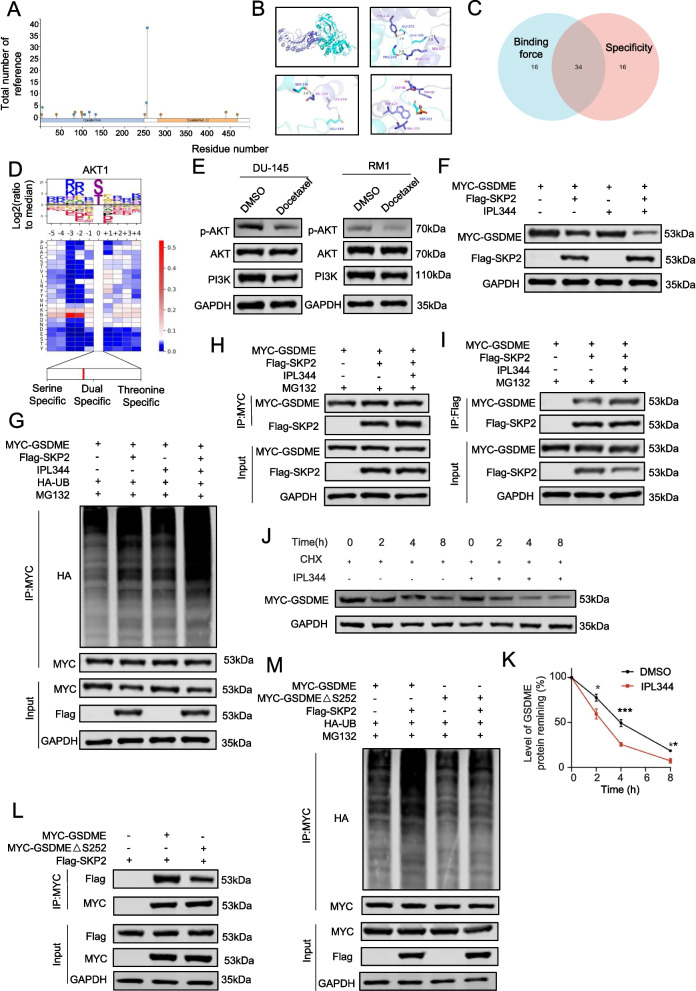
Phosphorylation at the S252 site leads to the degradation of GSDME. **A**: Phosphate-mapping site diagram of GSDME. **B**: Schematic diagrams depicting the potential binding motif of GSDME with SKP2 protein and its docking with mut GSDME and SKP2 (AlphaFold: AF-Q13309-F1). **C**: Overlapping Venn diagrams of the specific top 50 and binding top 50 kinases. **D**: Schematic diagram of AKT1 kinase binding. **E**: Protein expression of PI3K, AKT, and p-AKT before and after docetaxel treatment. **F**: GSDME protein expression after SKP2 overexpression with or without IPL344. **G**: Western blot analysis of ubiquitination levels after SKP2 overexpression with or without ILP. **H** and **I**: Co-IP analysis of SKP2 and GSDME binding with or without IPL344. **J** and **K**: Western blot analysis of GSDME protein expression in the presence or absence of IPL344 and CHX (50 μg/ml). **L**: Co-IP analysis of the binding of MYC-tagged WT GSDME or mutant GSDME with ectopically expressed FLAG-tagged SKP2 in 293 T cells treated with MG132. **M**: Western blot analysis of ubiquitination levels after SKP2 overexpression with MYC-tagged WT or mutant GSDME. Data are presented as mean ± SD (*n* = 3). n.s. not significant; *P < 0.01, **P < 0.01, ***P < 0.001

We designed a set of experiments in which the SKP2 gene was overexpressed under both docetaxel intervention and non-intervention conditions. The experimental results showed that the effect of docetaxel in promoting GSDME molecule expression was effectively inhibited by the overexpression of SKP2, further confirming the hypothesis that docetaxel regulates the expression level of GSDME through SKP2 (Supplementary Fig. 4 C). However, it is worth noting that docetaxel did not significantly affect the expression level of SKP2 itself, which led us to consider potential other regulatory pathways. SKP2 typically requires the phosphorylation of its targets for ubiquitination and subsequent degradation [20]. Utilizing the PhosphoSitePlus database, we predicted potential phosphorylation sites(https://www.phosphosite.org/homeAction) on GSDME and identified S252 and Y254 as significant (Fig. 5A). Computational simulations with Z-DOCK indicated increased pocket-binding stability upon mutation at S252 (Fig. 5B). Further analysis predicted AKT1 as a prominent kinase for S252, ranking within the top 50 for both binding ability and specificity, and previously associated with docetaxel (Fig. 5C, D).

Given the established role of the PI3K-AKT pathway in cancer and its inhibition by docetaxel [21–23], We confirmed the phosphorylation of GSDME at S252 using western blot experiments (Fig. 5E). Treatment with the AKT activator IPL-344 enhanced SKP2-mediated GSDME degradation (Fig. 5F) and reduced GSDME ubiquitination by SKP2 (Fig. 5G). Co-immunoprecipitation experiments further supported these findings, showing increased interaction between GSDME and SKP2 in the presence of IPL-344 (Fig. 5H, I). CHX experiments revealed accelerated GSDME degradation with IPL-344 treatment (Fig. 5J, K). Mutation of S252 weakened SKP2 binding to GSDME and reduced GSDME ubiquitination by SKP2 (Fig. 5L, M). Due to the constraints of objective conditions, we are currently unable to successfully synthesize a specific phosphorylation antibody targeting the S252 site of the GSDME molecule. Given this limitation, we referred to relevant literature in the field and conducted in vitro phosphorylation kinase experiments to achieve the detection of relevant indicators [24]. During the experiment, we first selected LPL344 as the intervention factor. The experimental results showed that after treatment with ILP344, the phosphorylation degree of the target molecule showed an upward trend (Supplementary Fig. 4D). To further verify this result and deeply explore the mechanism of the S252 site, we constructed a GST-tagged plasmid carrying the mutation at this site and conducted another in vitro phosphorylation kinase experiment. The experimental data indicated that when the S252 site mutated, the phosphorylation degree of the target molecule also decreased (Supplementary Fig. 4E).

These results underscore the regulatory role of AKT1-mediated phosphorylation at S252 in GSDME's interaction with SKP2.

### GSDME is closely related to the immune environment

**Fig. 6:**
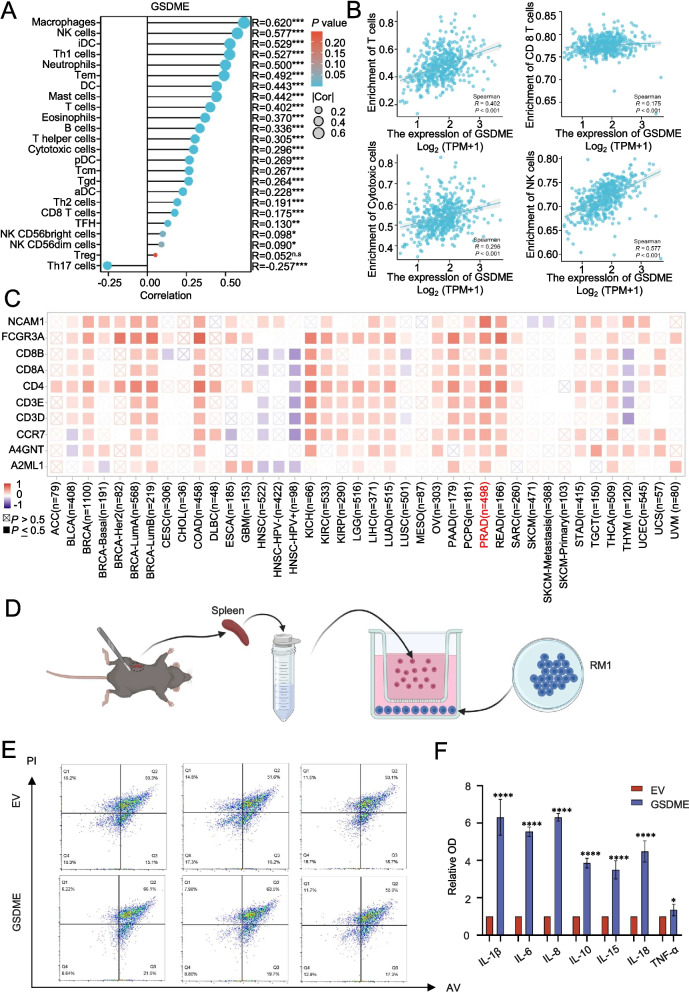
GSDME is closely related to the immune environment. **A**: Bubble map of GSDME in relation to immune cells in prostate cancer. **B**: Correlation diagram of the association between GSDME and immune cells in prostate cancer. **C**: Heatmap of the association between GSDME and important immune markers across cancers. **D**: Diagram of tumor and spleen cell co-culture. **E**: Representative images of tumor cell apoptosis after GSDME overexpression in the co-culture with spleen cells. **F**: OD values of cytokines in the cell supernatants. Data are presented as mean ± SD (*n* = 3). n.s., not significant; **P < 0.01, ***P < 0.001, ****P < 0.001

Our analysis of TCGA database revealed strong correlations between GSDME expression and various immune cell types in prostate tumors, particularly T lymphocytes, CD8 + lymphocytes, NK cells, and cytotoxic cells, which are essential for anti-tumor immune responses (Fig. 6A, B). GSDME showed higher correlation with immune cell surface markers in prostate tumors compared to other cancer types (Fig. 6C), consistent with pan-cancer analyses from TCGA (Supplementary Fig. 5 A).

Further investigation using GEO datasets (GSE70169) and TCGA data demonstrated that GSDME expression significantly influences immune cell populations and related immune cell expression (Supplementary Fig. 5B, C). These findings were supported by data from the TIMER database (Supplementary Fig. 5D).

Co-culture experiments using mouse spleen and PCa cells showed that cells overexpressing GSDME had a higher proportion of apoptosis after 48 h (Fig. 6D, E). ELISA analysis of the co-culture supernatant revealed increased levels of proinflammatory cytokines, such as IL-1β, IL-8, IL-10, IL-15, and IL-18, in the GSDME-overexpressing group (Fig. 6F). These results highlight the important role of GSDME in modulating immune responses in prostate tumors.

### GSDME affects the immune invasion of prostate tumors

**Fig. 7:**
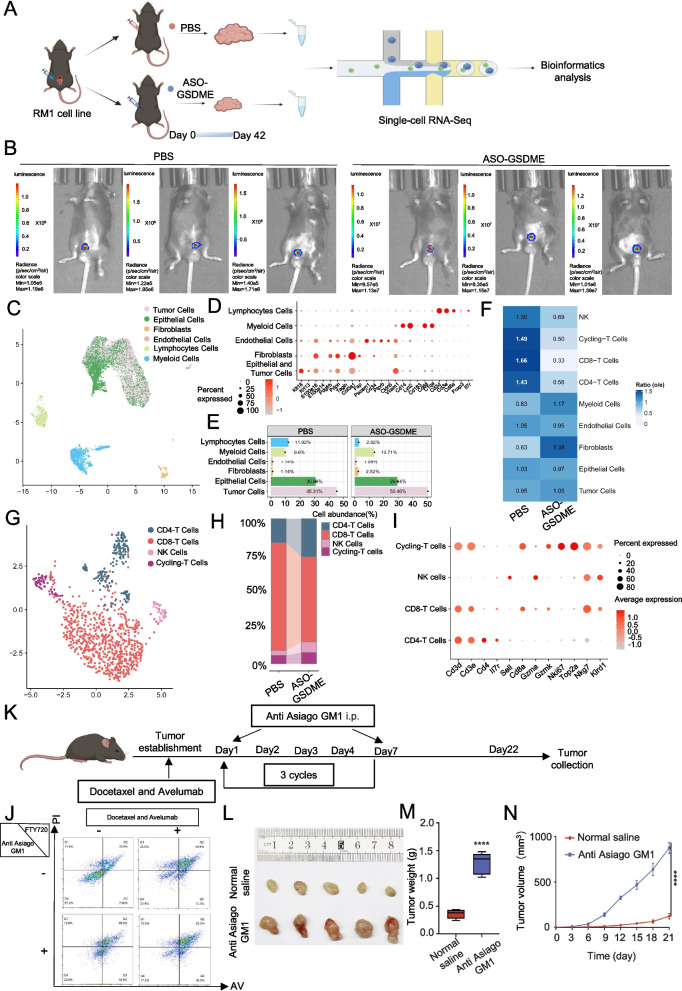
GSDME can affect the immune invasion of prostate tumors. **A**: Schematic representation of single-cell sequencing. **B**: Small animal imaging system showing the size of the prostate tumor in situ. **C**: Projection of UMAP annotated to six major cell types. **D**: Cluster marker genes. **E**: Bar plots showing the percentage (%) of cell types in experimental groups treated with PBS or ASO-GSDME. **F**: T cell tissue preference analysis. **G**: Projection of lymphocytes annotated to 4 major cell type groups. **H**: Bar plots showing the percentage (%) of cell types in the experimental groups treated with PBS or ASO-GSDME **I**: Cluster marker genes. **J**: Representative images of tumor cell apoptosis after drug intervention in co-culture with spleen cells. **K**: Schematic diagram of the mouse subcutaneous tumor model. **L**: Tumors from each group were harvested and photographed. **M**: Tumor weight in each group. **N**: Tumor volume at each time point. Data are presented as mean ± SD (*n* = 5). ****P < 0.0001

To further investigate the impact of GSDME on the prostate immune environment, we established an in situ prostate cancer model and divided the mice into two groups based on whether they received ASO-GSDME (Fig. 7A). In situ imaging of mice suggested that GSDME activation of the pyroptosis pathway could inhibit tumors to some extent (Fig. 7B).

The analysis revealed that the group treated with the drug had a greater proportion of immune lymphocytes than the control group (Fig. 7C and E). The genes used to label the clusters are shown as bubble maps (Fig. 7D). Tumor cells were identified by CNV clustering (Supplementary Fig. 6 A and B). Further analysis of the lymphocyte population revealed that in addition to the significant decrease in the number of CD8 + lymphocytes, the proportion of NK cells increased (Fig. 7G and H). However, notably, the proportion of NK cells in the administration group was still reduced (Fig. 7E), suggesting that GSDME knockdown has significant effects on both CD8 + T-cell and NK cell infiltration, and that the effect on CD8 + T cells may be more obvious. Given that avelumab targets T lymphocytes [25], we sought to determine the effect of NK cells on the effectiveness of combined immunotherapy. To explore this, we extracted mouse spleen cells and co-cultured tumor cells in combination with avelumab and docetaxel and simultaneously added FTY720 (lymphocyte inhibitor) or anti-Asiago GM1 (NK cell activity inhibitor) at the same time. The results showed that the immunosuppressive effect of FTY720 was not as strong as that of anti-Asiago GM1 (Fig. 7J and Supplement 6E).

In addition, we used an in vitro mouse model and treated it with GM1, an NK cell inhibitor (Fig. 7K). Tumor images, weights, and volume data were collected during the treatment period. The results revealed that inhibition of NK cell activity notably suppressed the efficacy of the combination of docetaxel and avelumab (Fig. 7L, M, and N).

These findings underscore the complex interplay among GSDME, immune cell dynamics, and therapeutic responses in prostate cancer, highlighting potential avenues for enhancing immunotherapy strategies by targeting NK cell-mediated immune responses. This observation was further supported by KEGG and GO analyses of the DEGs in Cluster 4 (Supplementary Figs. 6 C and D).

### GSDME plays an important role in enhancing the efficacy of docetaxel immunotherapy

**Fig. 8:**
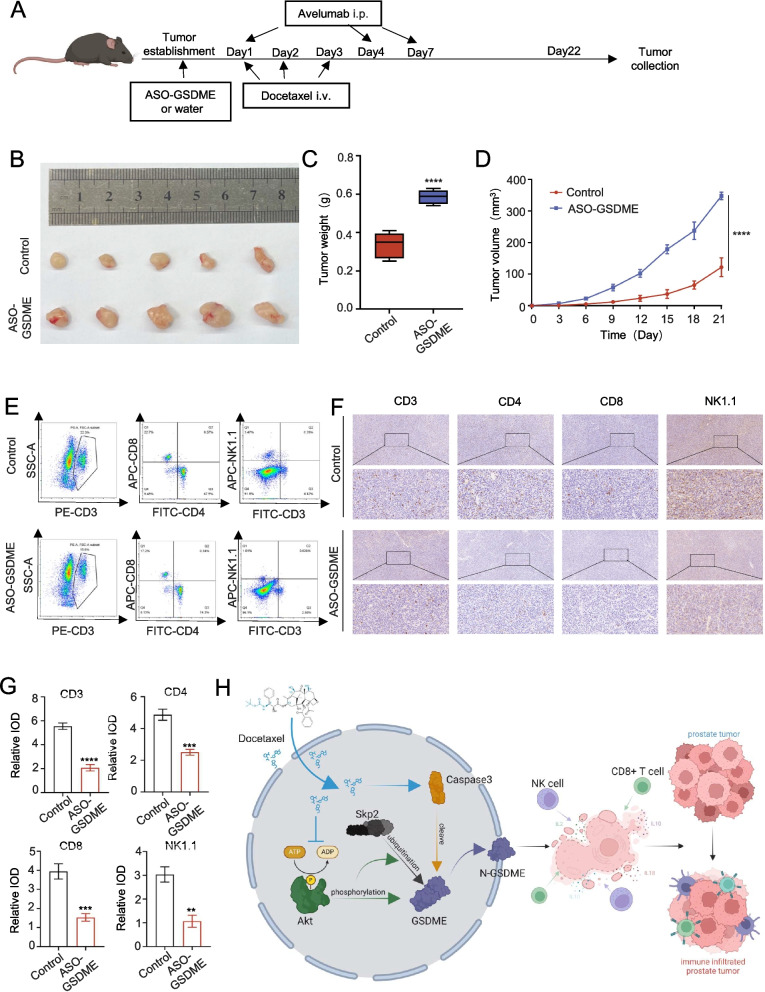
GSDME plays an important role in enhancing the efficacy of docetaxel immunotherapy. **A**: Schematic representation of the mouse subcutaneous tumor model. **B**: Tumors from each group were harvested and photographed. **C**: Tumor weight in each group. **D**: Tumor volume at each time point. **E**: Representative images of the immune lymphocytes types. **F**: Immunohistochemistry for immune-related indicators after GSDME knockdown. **G**: IOD value of each immune index after GSDME knockdown. **H**: A hypothesized model describing the mechanism by which docetaxel-induced GSDME pathway activation and pyroptosis enhance immunolethality in prostate cancer cells. Mechanistic data are presented as mean ± SD (*n* = 3). n.s., not significant; **P < 0.01, ***P < 0.001, ****P < 0.0001

To assess GSDME's role in combination therapies, we employed ASO-GSDME to knock down its expression (Fig. 8A). Tumor progression was monitored via imaging, weight, and volume measurements. GSDME knockdown moderately decreased combination therapy efficacy compared to controls (Fig. 8B-D).

Flow cytometry analysis of mouse subcutaneous tumors showed reduced levels of CD4 + T cells, CD8 + T cells, and NK cells in the ASO-GSDME group (Fig. 8E). Immunohistochemical staining further confirmed decreased CD3, CD4, CD8, CD49b, and NK1.1 expression in this group relative to the combination therapy group (Fig. 8F-G).

In conclusion, our results indicate that docetaxel inhibits the PI3K‒AKT pathway, reducing SKP2 binding to GSDME and increasing GSDME expression, which enhances the immune response (Fig. 8H). These findings underscore the potential of targeting GSDME to improve prostate cancer combination therapies.

## Discussion

Given the generally low immune mutation load observed in prostate cancer, this condition has been classified as a "cold tumor" in terms of immunotherapy responsiveness. This classification underscores the challenge of achieving significant therapeutic effects with standalone immunotherapy approaches for prostate cancer treatment [26]. Therefore, researchers have shifted their focus toward combination therapies involving PD-1 inhibitors to explore and enhance therapeutic efficacy. Clinical trials have demonstrated that nivolumab combined with docetaxel exhibits clinical activity in chemotherapy-naïve patients with metastatic castration-resistant prostate cancer (mCRPC) [27]. The results obtained in this study closely align with our observations in the C57 mouse model (Fig. 2). This promising outcome strongly indicates that further exploration of its mechanisms has substantial potential to significantly impact the field of prostate cancer immunotherapy.

Docetaxel is widely used as a chemotherapeutic agent in lung and breast cancer treatment regimens. Numerous studies have examined its effect on immune lymphocytes [10, 11]. According to previous studies, after docetaxel chemotherapy, various immune-related lymphocyte subsets, such as CD3 + and CD3 + CD4 + cells, CD4 +/CD8 + ratio, and NK cells, increase to varying degrees. This finding was consistent with the clinical data collected at our hospital, providing robust clinical evidence (Fig. 1). Building upon these findings, we conducted further exploratory research to analyze immune-associated lymphocytes in patients with prostate cancer after chemotherapy. Comparative analysis revealed that compared with pre-chemotherapy levels, there was an increase in the numbers of CD3 + and CD3 + CD4 + cells, the CD4 +/CD8 + ratio, and the number of NK cells in prostate cancer patients. These results mirrored those of puncture samples collected after chemotherapy (Fig. 1). These findings suggest that docetaxel can modify the immune environment of patients with prostate cancer, potentially enhancing the effectiveness of immunotherapy. This conclusion aligns with the results of previous research in this field [28].

Docetaxel primarily exerts its pharmacological effects by promoting apoptosis through its action on microtubules. As part of the taxane class of chemotherapy drugs, docetaxel targets microtubules, which are crucial components of the cytoskeleton involved in vital cellular processes, such as cell division [29]. Apoptosis is a form of programmed cell death in which cells undergo controlled degradation, allowing them to maintain their internal stability. By contrast, pyroptosis induces inflammation and disrupts the internal environment [30]. Recent studies have demonstrated a clear shift in the mode of cell death from apoptosis to pyroptosis induced by chemotherapeutic drugs, which is primarily mediated by GSDME molecules [9]. Notably, this phenomenon was also observed in prostate cancer cell lines (Fig. 2). Therefore, we hypothesized that docetaxel may promote pyroptosis to a certain extent, subsequently increasing immune infiltration, thereby improving the overall effectiveness of immunotherapy.

In our study, docetaxel treatment significantly altered the expression of GSDME at the protein level without affecting its transcription. These findings led us to hypothesize that SKP2 molecules might regulate this process. SSKP2 typically requires prior phosphorylation of its target protein to initiate degradation [20]. Through bioinformatics analysis and experimental validation, we discovered that AKT1 facilitated the phosphorylation of GSDME at S252. Numerous studies have reported that docetaxel inhibits the activation of the PI3K-AKT pathway [21, 31, 32]. Based on these findings, we propose a molecular mechanism by which docetaxel regulates the expression of GSDME. Docetaxel inhibits the PI3K‒AKT pathway, thereby reducing the phosphorylation of the GSDME molecule. This reduction in phosphorylation prevented GSDME from being degraded by SKP2.

Notably, single-cell sequencing revealed that GSDME-mediated pyroptosis influences immune cell infiltration, resulting in significant changes not only in CD8 + lymphocytes but also in the proportion of NK cells (Fig. 7F). Avelumab, a PD-1 inhibitor that primarily targets T lymphocyte responses, has already been used in urothelial carcinoma [33]. However, we observed that inhibiting NK cell activity could partially diminish the effectiveness of combination therapy, highlighting a complex regulatory mechanism within the immune microenvironment. Previous reports have suggested that enhancing this effect may involve the addition of multiple NK ligands [34]. We hypothesized that the release of pyrogenic cytokines might impact NK cell function; however, the specific pathways through which this molecule affects NK cell differentiation, infiltration, and cytotoxicity require further investigation. However, the specific pathways and regulatory mechanisms underlying this phenomenon require further investigation.

However, this study has several limitations. The number of patients eligible for standard prostate cancer chemotherapy from whom we could collect clinical specimens was limited, resulting in a relatively low level of evidence. In addition, AKT-mediated phosphorylation activates the SCF complex and E3 ligase. Exploring these phosphorylation events in more detail could provide deeper insight into the regulatory mechanisms involved [35].

## Conclusions

In conclusion, this study clarified the effects of GSDME on immunotherapy and its mechanism of action. Elucidating the influence of docetaxel. The specific mechanism of GSDME expression regulation, the specific sites of its ubiquitination, and the essence of its expression changes were confirmed by a fine regulatory mechanism. Docetaxel changes the immune microenvironment of prostate cancer by affecting the AKT-SKP2-GSDME signaling axis. A new approach to enhance the efficacy of immunotherapy in prostate cancer has been explored.

## Supplementary Information

Supplementary Material 1: Supplementary Fig 1. Docetaxel affects immune lymphocytes changes. A: Proportion of immune lymphocytes during non-small cell lung cancer and breast cancer chemotherapy cycle. B: The overlapping Venn diagrams of immune-related genes and docetaxel influencing genes. C: Relationship between related genes and immune cells in prostate tumors. n.s., no significance; *P < 0.05, ***P < 0.001,****P < 0.0001. Supplementary Fig 2. Docetaxel can induce a shift in the mode of death of prostate cancer cells. A: Schematic diagram of a mouse subcutaneous tumor model. B: Survival curves of mice in each group. Data represented as mean ± SD (*n* = 3). C: Experiment of Cell Plate Cloning under Drug Intervention; D: Quantitative Statistics of Colony Formation in Plate Cloning Experiment. Data represented as mean ± SD (*n* = 3). n.s., no significance; **P < 0.01, ***P < 0.001, ****P < 0.0001. Supplementary Fig 3. Docetaxel can induce a shift in the mode of death of prostate cancer cells. A: mRNA expression levels of GSDME in prostate cancer cell lines from HPA. B: protein expression levels of GSDME in prostate cancer cell lines. C: Changes of protein expression of Caspase1 and Caspase3 before and after docetaxel treatment D: mRNA expression levels of GSDME after GSDME overexpression. E: protein expression levels of GSDME after GSDME overexpression. F: CCK8 assay on the OD value in 96-well plates of the RM1 cells and DU145 cells after GSDME overexpression. G: Colony formation assay on colony numbers of the RM1 cells and DU145 cells after GSDME overexpression. Data represented as mean ± SD (*n* = 3). n.s., no significance;**P < 0.01, ***P < 0.001, ****P < 0.0001. Supplementary Fig 4. SKP2 ubiquitinates GSDME and promotes its degradation. A: protein expression levels of GSDME after adding different drugs. B: protein expression levels of GSDME in the condition of docetaxel and TFA. C: protein expression levels of GSDME in the condition of docetaxel and SKP2. D: The degree of molecular phosphorylation of GSDME under the intervention of IPL34. E: The degree of phosphorylation of the GSDME molecule after the mutation at the S252 position. Supplementary Fig 5. GSDME is closely related to immune environment. A: Relationship between GSDME segmentation and immune cells in pan-carcinoma. B: Enrichment analysis of GSDME overexpression data sets. C: Effect of differential expression of GSDME on immune cell expression. D: GSDME molecules are associated with immune cell infiltration from timer datesets. Supplementary Fig 6. GSDME can affect the immune invasion of prostate tumors. A: Different cells are subdistributed on chromosomes. B: Time sequence analysis of different cell subpopulations. C: Lymphocyte grouping affects functional changes D: Specific lymphocyte cluster enrichment function. E: Proportion of surviving tumor cells. Mechanism diagram Data represented as mean ± SD (*n* = 3). n.s, no significance, *P < 0.05,**P < 0.01. Supplementary Table 1. Oligonucleotides used for relative gene expression by qRT-PCR. Supplementary Table 2. The oligonucleotides of siRNA or shRNA. *The oligonucleotides from WeizhenBio, Shandong, China.

Supplementary Material 2. 

## Data Availability

The datasets used and analysed during the current study are available from the corresponding author on reasonable request.

## Abbreviations

PCa: Prostate cancer
TME: Tumor microenvironment
GSDME: Gasdermin E
IHC: Immunohistochemistry
RT-qPCR: Real-time quantitative polymerase chain reaction
WB: Western Blot
