# Supplementary material for: Docetaxel induced activation of GSDME pathway and pyroptosis enhance immune lethality in prostate cancer cells
Source: J Exp Clin Cancer Res. 2025 Dec 18;45:22. doi: 10.1186/s13046-025-03614-1 (PMC12825250; doi:10.1186/s13046-025-03614-1)
Supplement: Supplementary file 1 — Supplementary Material 1: Supplementary Fig 1. Docetaxel affects immune lymphocytes changes. A: Proportion of immune lymphocytes during non-small cell lung cancer and breast cancer chemotherapy cycle. B: The overlapping Venn diagrams of immune-related genes and docetaxel influencing genes. C: Relationship between related genes and immune cells in prostate tumors. n.s., no significance; *P < 0.05, ***P < 0.001,****P < 0.0001. Supplementary Fig 2. Docetaxel can induce a shift in the mode of death of prostate cancer cells. A: Schematic diagram of a mouse subcutaneous tumor model. B: Survival curves of mice in each group. Data represented as mean ± SD (n = 3). C: Experiment of Cell Plate Cloning under Drug Intervention; D: Quantitative Statistics of Colony Formation in Plate Cloning Experiment. Data represented as mean ± SD (n = 3). n.s., no significance; **P < 0.01, ***P < 0.001, ****P < 0.0001. Supplementary Fig 3. Docetaxel can induce a shift in the mode of death of prostate cancer cells. A: mRNA expression levels of GSDME in prostate cancer cell lines from HPA. B: protein expression levels of GSDME in prostate cancer cell lines. C: Changes of protein expression of Caspase1 and Caspase3 before and after docetaxel treatment D: mRNA expression levels of GSDME after GSDME overexpression. E: protein expression levels of GSDME after GSDME overexpression. F: CCK8 assay on the OD value in 96-well plates of the RM1 cells and DU145 cells after GSDME overexpression. G: Colony formation assay on colony numbers of the RM1 cells and DU145 cells after GSDME overexpression. Data represented as mean ± SD (n = 3). n.s., no significance;**P < 0.01, ***P < 0.001, ****P < 0.0001. Supplementary Fig 4. SKP2 ubiquitinates GSDME and promotes its degradation. A: protein expression levels of GSDME after adding different drugs. B: protein expression levels of GSDME in the condition of docetaxel and TFA. C: protein expression levels of GSDME in the condition of docetaxel and SKP2. D: The degree [file 13046_2025_3614_MOESM1_ESM.zip › Supplementary material/supplement.docx]

**Docetaxel induced activation of GSDME pathway and pyroptosis enhance immune lethality in prostate cancer cells**

Ruoyang Liu, Long Zhang, Guoqing Xie, Xiang Li, Yu Liu, Ningyang Li, Aravind Raveendran, Yuankang Feng, Fubo Lu, Xiyue Deng, Junyi Li, Jinjian Yang, Zhenlin Huang, Zhankui Jia

**Files included in the supplementary information**

**Supplementary Fig 1 （Supplemental to Fig. 1）** Docetaxel affects immune lymphocyte changes.

**Supplementary Fig 2 （Supplemental to Fig.2）**Docetaxel can induce a shift in the mode of death of prostate cancer cells.

**Supplementary Fig 3 （Supplemental to Fig.2）**Docetaxel can induce a shift in the mode of death of prostate cancer cells.

**Supplementary Fig 4 （Supplemental to Fig. 3 and 5）**SKP2 ubiquitinates GSDME and promotes its degradation.

**Supplementary Fig 5（Supplemental to Fig. 6）**GSDME is closely related to immune environment.

**Supplementary Fig 6（Supplemental to Fig. 7）**GSDME can affect the immune invasion of prostate tumors.

**Supplementary Table1**. Oligonucleotides used for relative gene expression by qRT-PCR.

**Supplementary Table 2**. The oligonucleotides of sh- RNAs or ASO-GSDME.

**Supplement figure1**

**
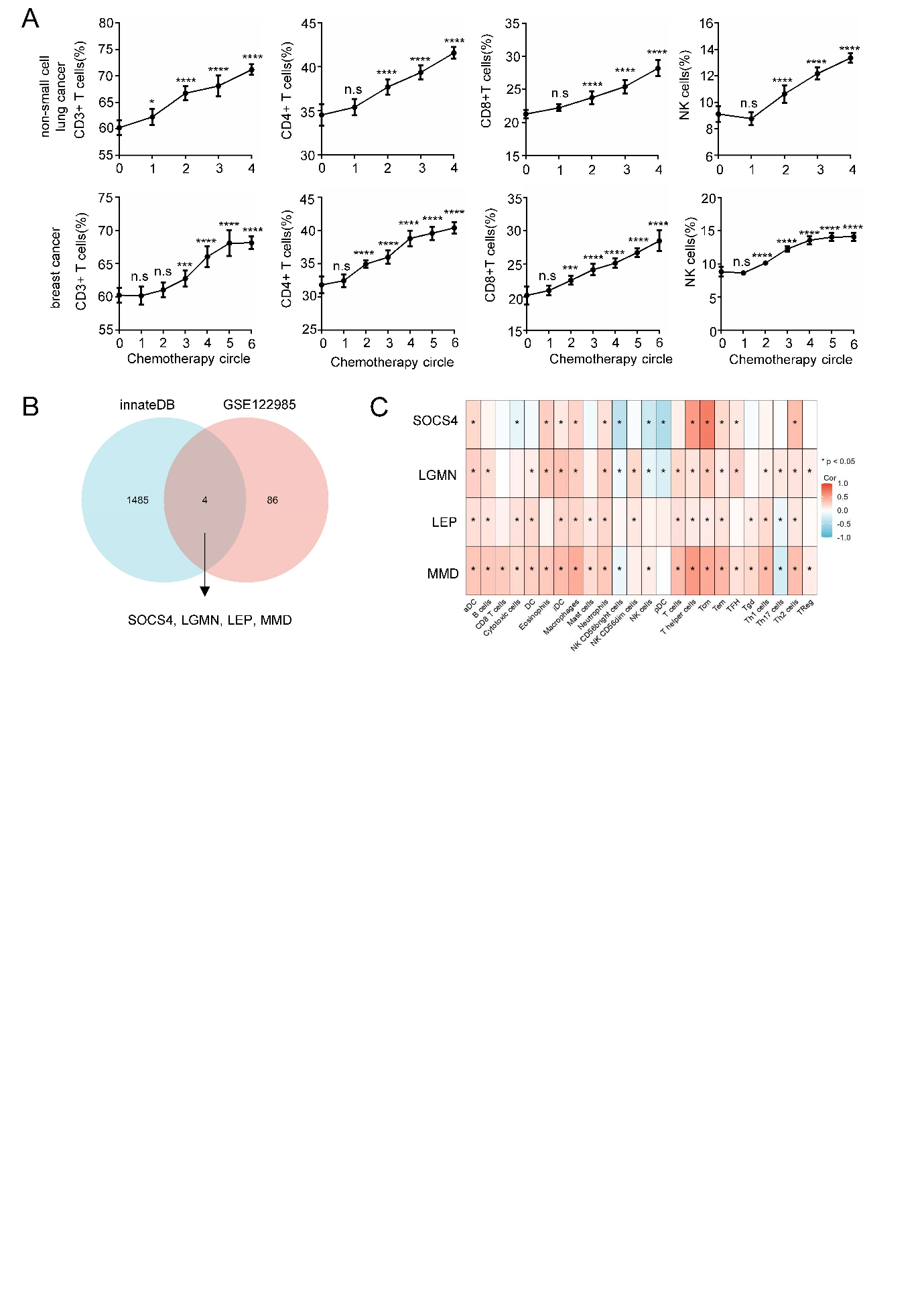
**

**Supplementary Fig 1. Docetaxel affects immune lymphocyte changes.**

A: Proportion of immune lymphocytes during non-small cell lung cancer and breast cancer chemotherapy cycle. B: The overlapping Venn diagrams of immune-related genes and docetaxel influencing genes. C: Relationship between related genes and immune cells in prostate tumors. n.s., no significance; *P < 0.05, ***P < 0.001, ****P < 0.0001

**Supplement figure2**

**
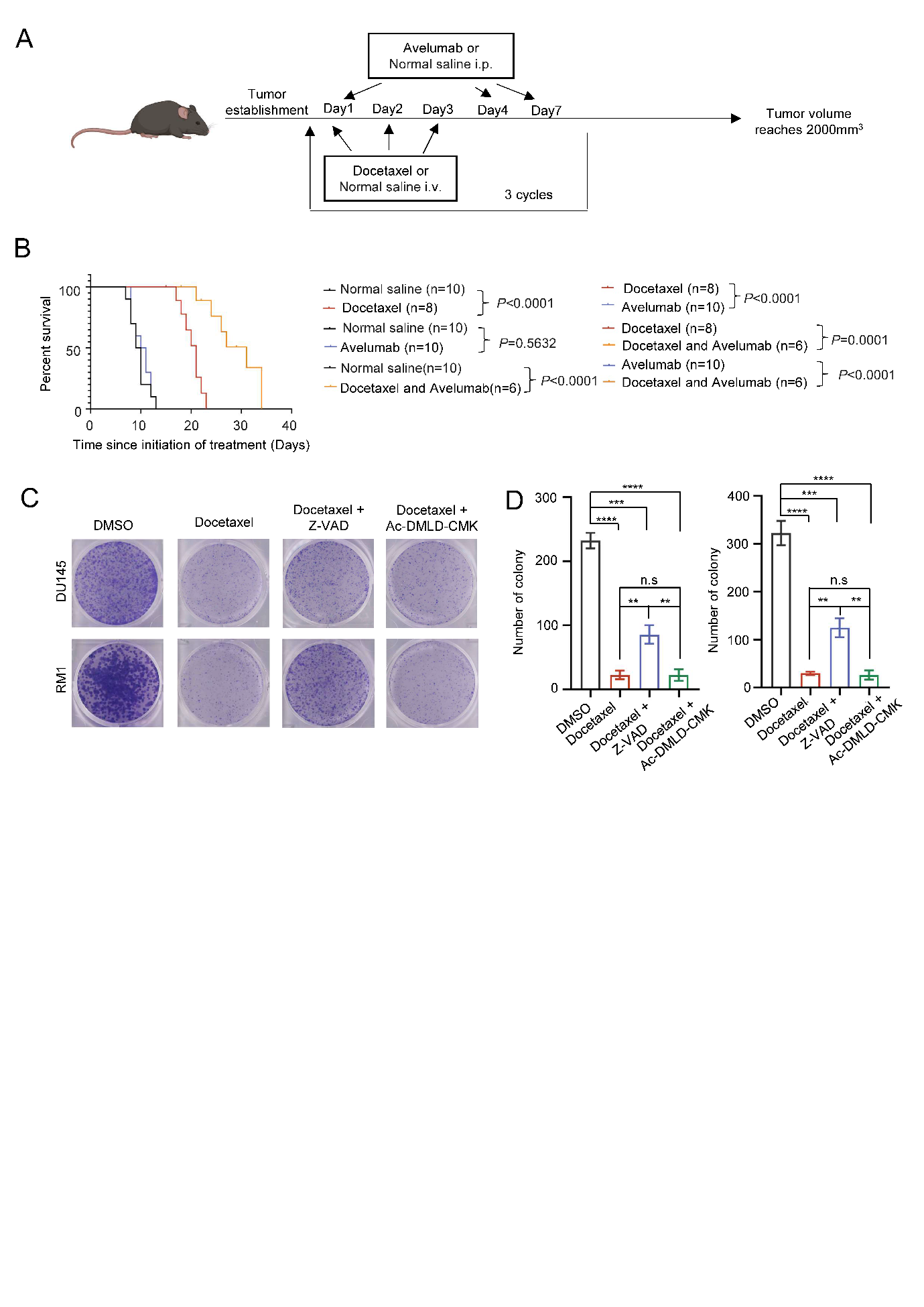
**

**Supplementary Fig 2. Docetaxel can induce a shift in the mode of death of prostate cancer cells.** A: Schematic diagram of a mouse subcutaneous tumor model. B: Survival curves of mice in each group. Data represented as mean ± SD (n = 3).

C: Experiment of Cell Plate Cloning under Drug Intervention; D: Quantitative Statistics of Colony Formation in Plate Cloning Experiment. Data represented as mean ± SD (n = 3). n.s., no significance; **P < 0.01, ***P < 0.001, ****P < 0.0001

**Supplement figure3**


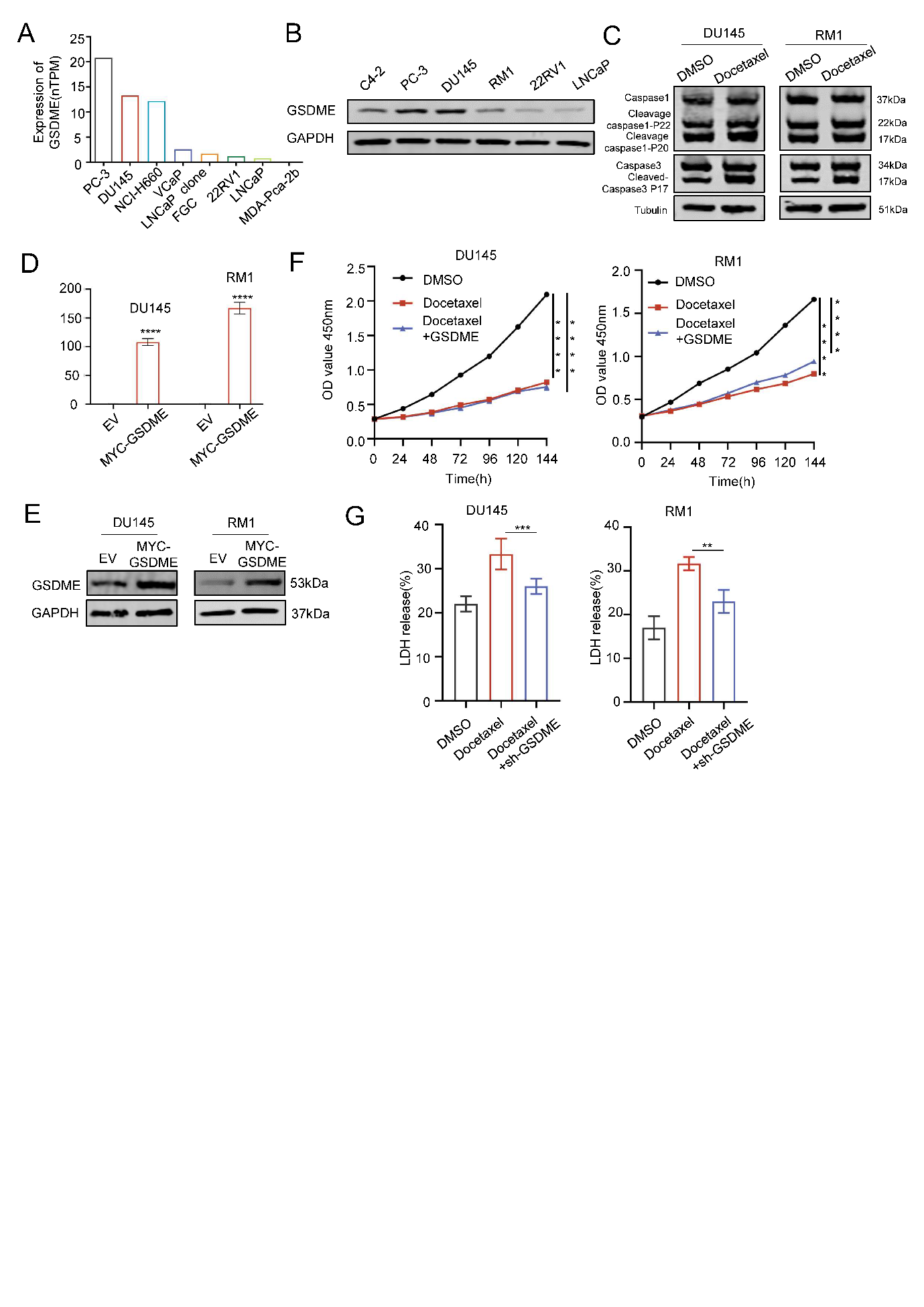


**Supplementary Fig 3. Docetaxel can induce a shift in the mode of death of prostate cancer cells**. A: mRNA expression levels of GSDME in prostate cancer cell lines from HPA. B: protein expression levels of GSDME in prostate cancer cell lines. C: Changes of protein expression of Caspase1 and Caspase3 before and after docetaxel treatment D: mRNA expression levels of GSDME after GSDME overexpression. E: protein expression levels of GSDME after GSDME overexpression. F: CCK8 assay on the OD value in 96-well plates of the RM1 cells and DU145 cells after GSDME overexpression. G: Colony formation assay on colony numbers of the RM1 cells and DU145 cells after GSDME overexpression. Data represented as mean ± SD (n = 3). n.s., no significance; **P < 0.01, ***P < 0.001, ****P < 0.0001

**Supplement figure4**

**
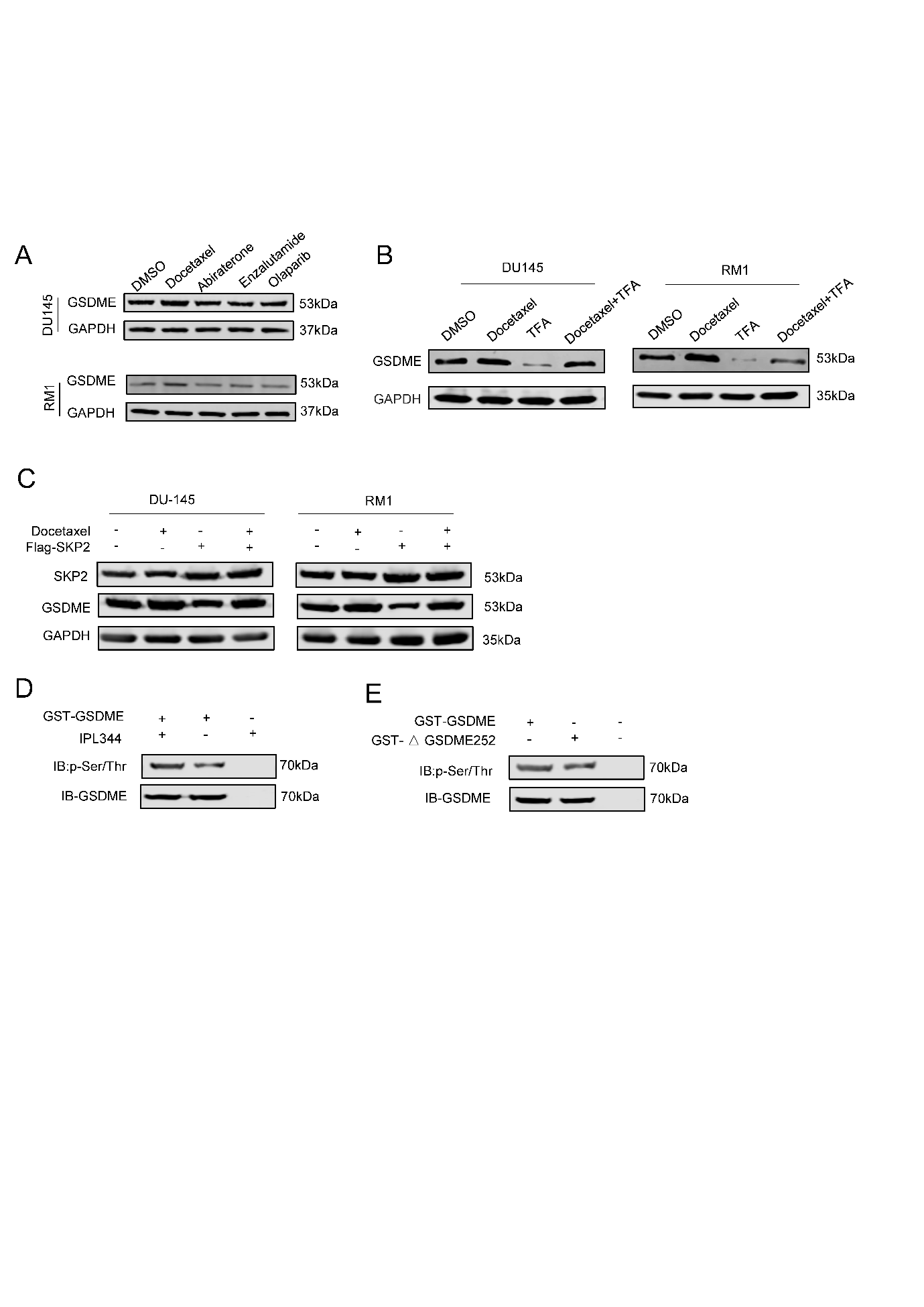
**

**Supplementary Fig 4. SKP2 ubiquitinates and modifies GSDME and promotes its degradation.** A: protein expression levels of GSDME after adding different drugs.

B: protein expression levels of GSDME in the condition of docetaxel and TFA. C: protein expression levels of GSDME in the condition of docetaxel and SKP2. D: The degree of molecular phosphorylation of GSDME under the intervention of IPL34. E: The degree of phosphorylation of the GSDME molecule after the mutation at the S252 position

**Supplement figure5**


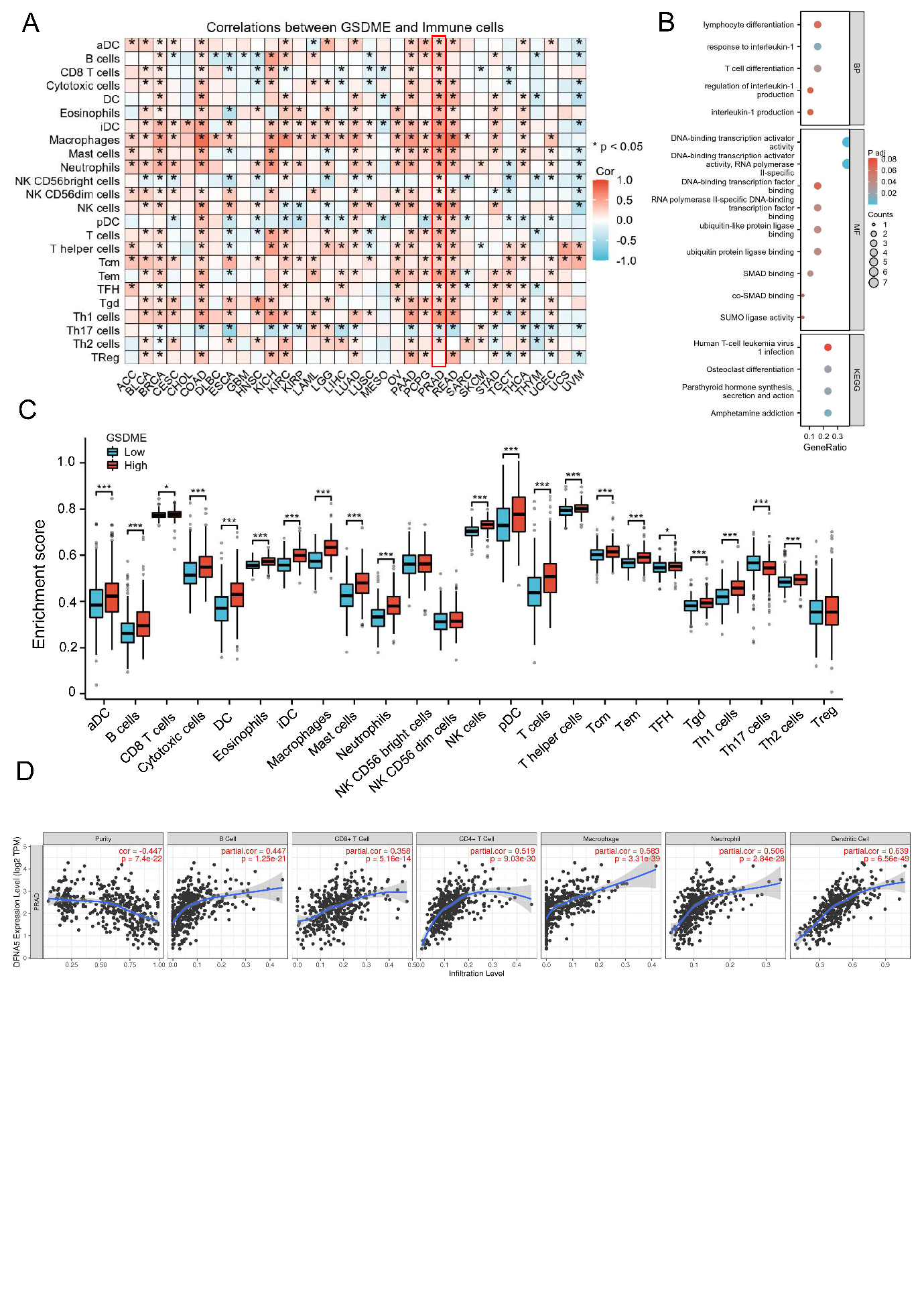


**Supplementary Fig 5. GSDME is closely related to immune environment.** A: Relationship between GSDME segmentation and immune cells in pan-carcinoma. B: Enrichment analysis of GSDME overexpression data sets. C: Effect of differential expression of GSDME on immune cell expression. D: GSDME molecules are associated with immune cell infiltration from timer datesets.

**Supplement figure6**


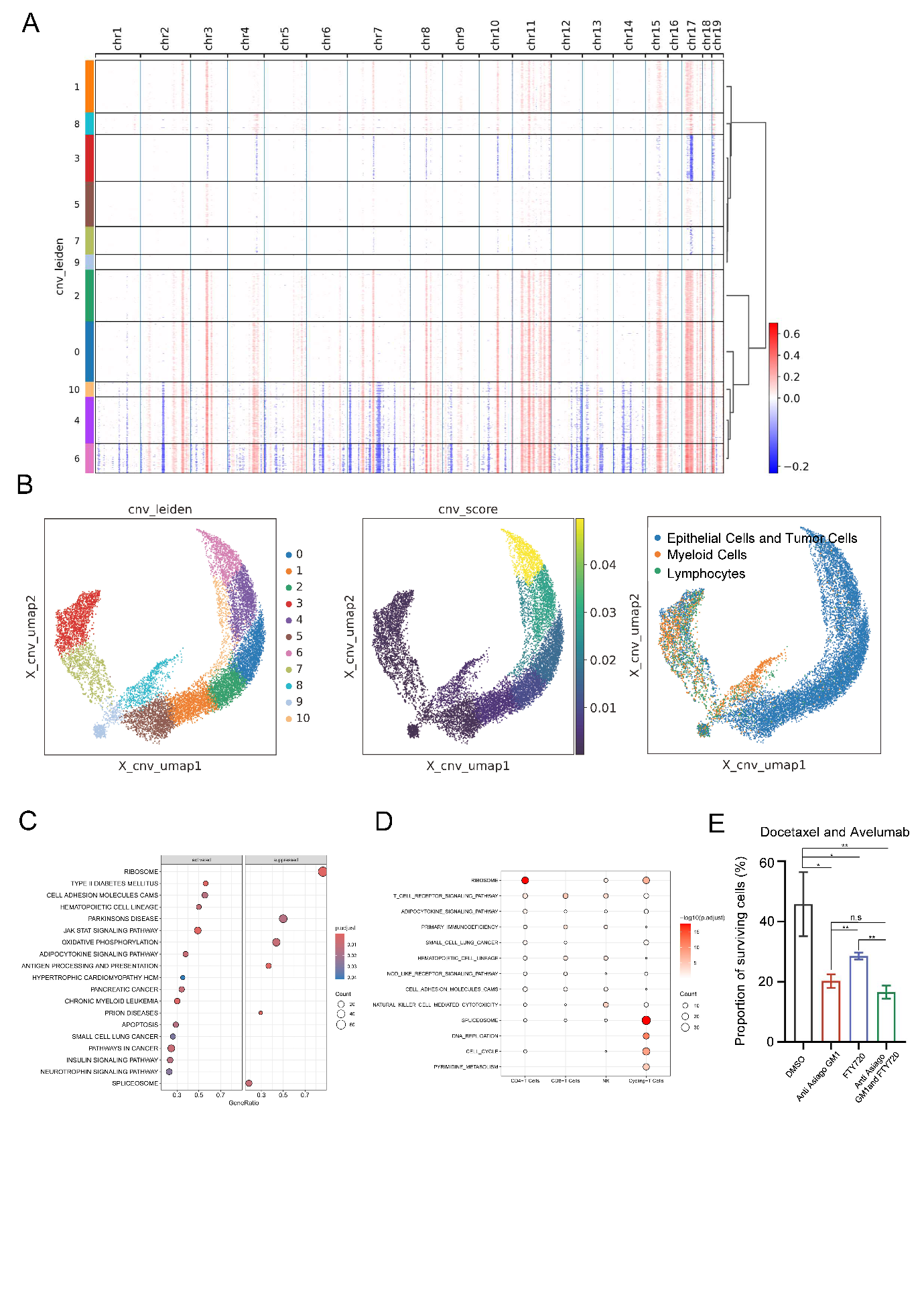


**Supplementary Fig 6.** **GSDME can affect the immune invasion of prostate tumors.** A: Different cells are subdistributed on chromosomes. B: Time sequence analysis of different cell subpopulations. C: Lymphocyte grouping affects functional changes D: Specific lymphocyte cluster enrichment function. E: Proportion of surviving tumor cells. Mechanism diagram Data represented as mean ± SD (n = 3). n.s, no significance, **P* < 0.05,***P* < 0.01

**Supplementary Table 1. Oligonucleotides used for relative gene expression by qRT-PCR.**

| Target gene | Forward primers (5’→3’) | Reverse primers (5’ →3’) |
| --- | --- | --- |
| GSDME | CGGGCGCGCGGATAAT | GTCTCTGCCAGCACCAGAAT |

**Supplementary Table 2.** **The oligonucleotides of siRNA or shRNA.**

| Product name | Forward primers (5’-3’) |
| --- | --- |
| GSDME shRNA-1 | GGTGACCTGATTGCAGTATCA |
| GSDME shRNA-2 | GGAGTCGGACTTTGTGAAATA |
| GSDME shRNA-3 | GGCGGTCCTATTTGATGATGA |
| ASO-GSDME-1 | C*T*C*A*T*G*C*T*C*G*A*A*G*C*C*A*C*C*A*T |
| ASO-GSDME-2 | C*A*C*C*A*G*C*C*A*C*A*T*C*A*T*C*A*C*A*C |
| ASO-GSDME-3 | C*C*T*T*T*G*C*C*A*C*C*A*A*C*G*T*T*C*A*G |

* The oligonucleotides from WeizhenBio, Shandong, China.
