# Supplementary figures and images for: Docetaxel induced activation of GSDME pathway and pyroptosis enhance immune lethality in prostate cancer cells
Source: J Exp Clin Cancer Res. 2025 Dec 18;45:22. doi: 10.1186/s13046-025-03614-1 (PMC12825250; doi:10.1186/s13046-025-03614-1)

# B

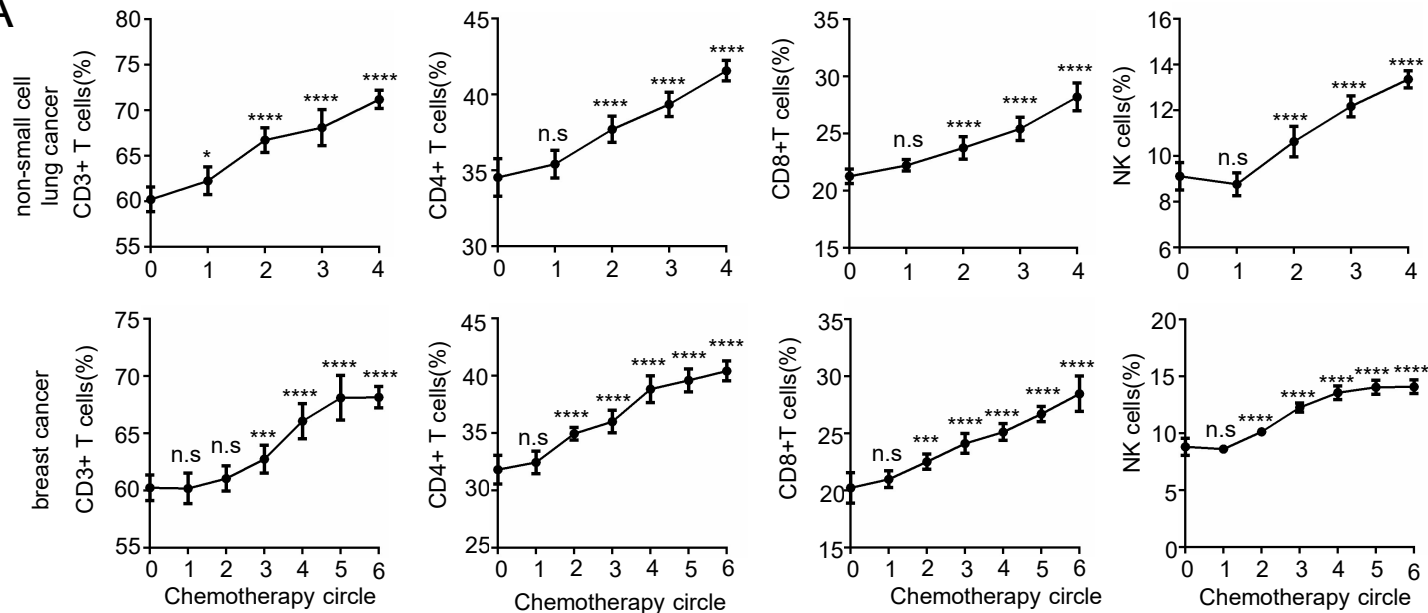

# B

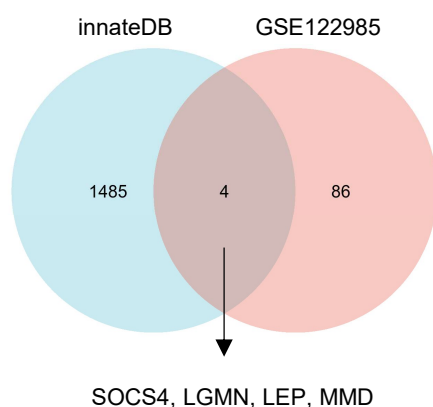

C

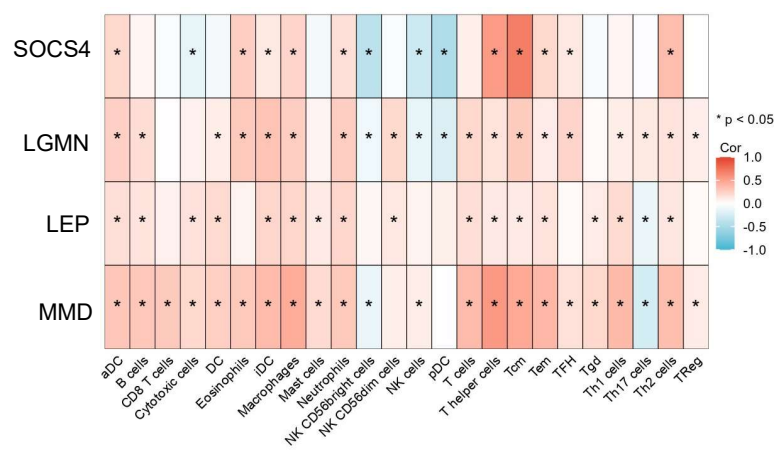

Supplement: Supplementary file 1 — Supplementary Material 1: Supplementary Fig 1. Docetaxel affects immune lymphocytes changes. A: Proportion of immune lymphocytes during non-small cell lung cancer and breast cancer chemotherapy cycle. B: The overlapping Venn diagrams of immune-related genes and docetaxel influencing genes. C: Relationship between related genes and immune cells in prostate tumors. n.s., no significance; *P < 0.05, ***P < 0.001,****P < 0.0001. Supplementary Fig 2. Docetaxel can induce a shift in the mode of death of prostate cancer cells. A: Schematic diagram of a mouse subcutaneous tumor model. B: Survival curves of mice in each group. Data represented as mean ± SD (n = 3). C: Experiment of Cell Plate Cloning under Drug Intervention; D: Quantitative Statistics of Colony Formation in Plate Cloning Experiment. Data represented as mean ± SD (n = 3). n.s., no significance; **P < 0.01, ***P < 0.001, ****P < 0.0001. Supplementary Fig 3. Docetaxel can induce a shift in the mode of death of prostate cancer cells. A: mRNA expression levels of GSDME in prostate cancer cell lines from HPA. B: protein expression levels of GSDME in prostate cancer cell lines. C: Changes of protein expression of Caspase1 and Caspase3 before and after docetaxel treatment D: mRNA expression levels of GSDME after GSDME overexpression. E: protein expression levels of GSDME after GSDME overexpression. F: CCK8 assay on the OD value in 96-well plates of the RM1 cells and DU145 cells after GSDME overexpression. G: Colony formation assay on colony numbers of the RM1 cells and DU145 cells after GSDME overexpression. Data represented as mean ± SD (n = 3). n.s., no significance;**P < 0.01, ***P < 0.001, ****P < 0.0001. Supplementary Fig 4. SKP2 ubiquitinates GSDME and promotes its degradation. A: protein expression levels of GSDME after adding different drugs. B: protein expression levels of GSDME in the condition of docetaxel and TFA. C: protein expression levels of GSDME in the condition of docetaxel and SKP2. D: The degree [file 13046_2025_3614_MOESM1_ESM.zip › Supplementary material/supplement figure1.pdf]

**A**

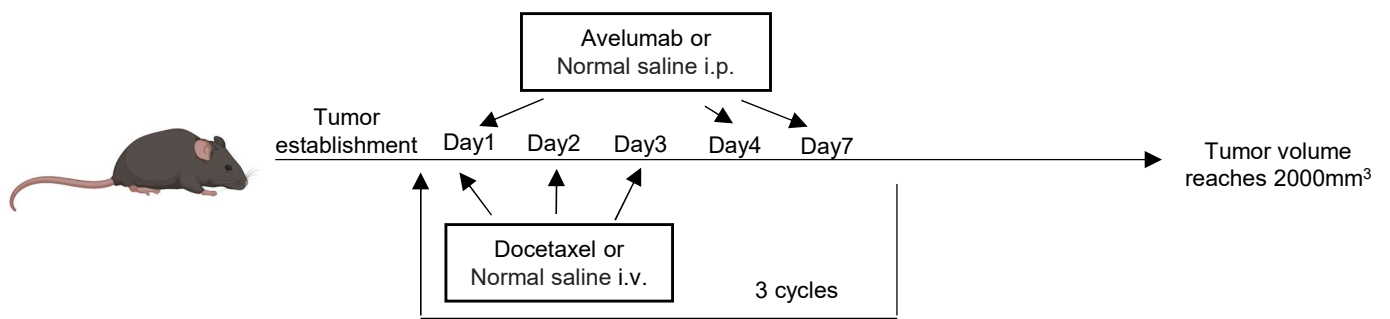

**B**

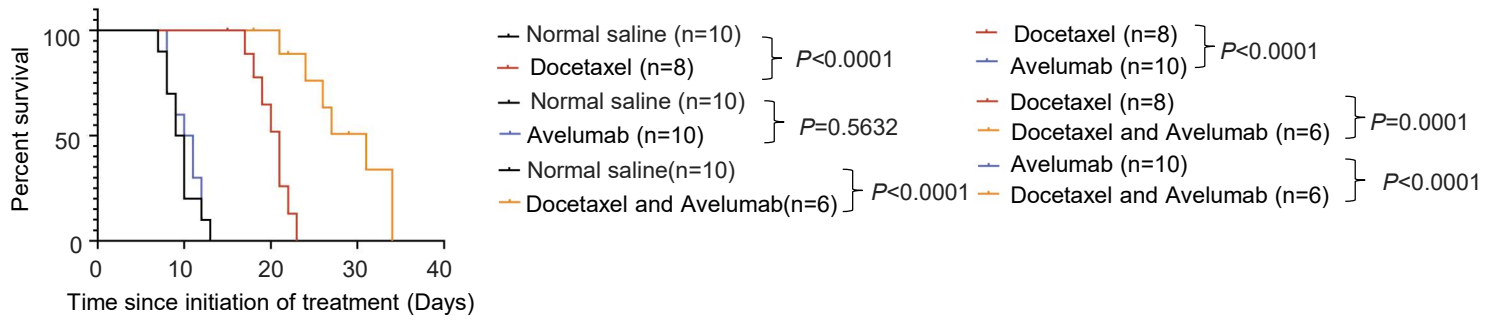

**C**

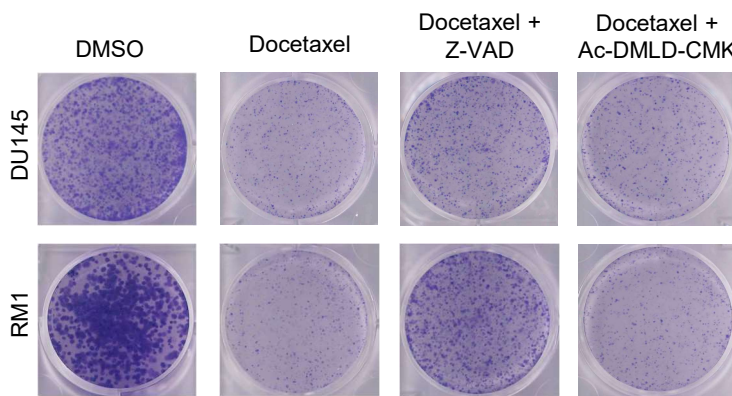

**D**

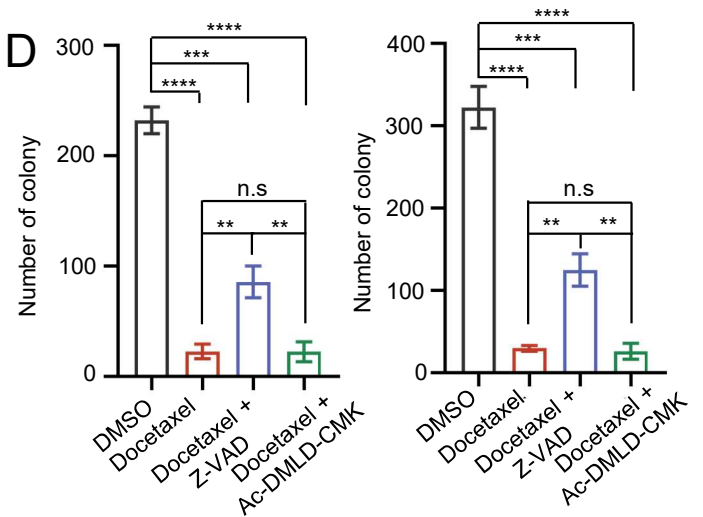

Supplement: Supplementary file 1 — Supplementary Material 1: Supplementary Fig 1. Docetaxel affects immune lymphocytes changes. A: Proportion of immune lymphocytes during non-small cell lung cancer and breast cancer chemotherapy cycle. B: The overlapping Venn diagrams of immune-related genes and docetaxel influencing genes. C: Relationship between related genes and immune cells in prostate tumors. n.s., no significance; *P < 0.05, ***P < 0.001,****P < 0.0001. Supplementary Fig 2. Docetaxel can induce a shift in the mode of death of prostate cancer cells. A: Schematic diagram of a mouse subcutaneous tumor model. B: Survival curves of mice in each group. Data represented as mean ± SD (n = 3). C: Experiment of Cell Plate Cloning under Drug Intervention; D: Quantitative Statistics of Colony Formation in Plate Cloning Experiment. Data represented as mean ± SD (n = 3). n.s., no significance; **P < 0.01, ***P < 0.001, ****P < 0.0001. Supplementary Fig 3. Docetaxel can induce a shift in the mode of death of prostate cancer cells. A: mRNA expression levels of GSDME in prostate cancer cell lines from HPA. B: protein expression levels of GSDME in prostate cancer cell lines. C: Changes of protein expression of Caspase1 and Caspase3 before and after docetaxel treatment D: mRNA expression levels of GSDME after GSDME overexpression. E: protein expression levels of GSDME after GSDME overexpression. F: CCK8 assay on the OD value in 96-well plates of the RM1 cells and DU145 cells after GSDME overexpression. G: Colony formation assay on colony numbers of the RM1 cells and DU145 cells after GSDME overexpression. Data represented as mean ± SD (n = 3). n.s., no significance;**P < 0.01, ***P < 0.001, ****P < 0.0001. Supplementary Fig 4. SKP2 ubiquitinates GSDME and promotes its degradation. A: protein expression levels of GSDME after adding different drugs. B: protein expression levels of GSDME in the condition of docetaxel and TFA. C: protein expression levels of GSDME in the condition of docetaxel and SKP2. D: The degree [file 13046_2025_3614_MOESM1_ESM.zip › Supplementary material/supplement figure2.pdf]

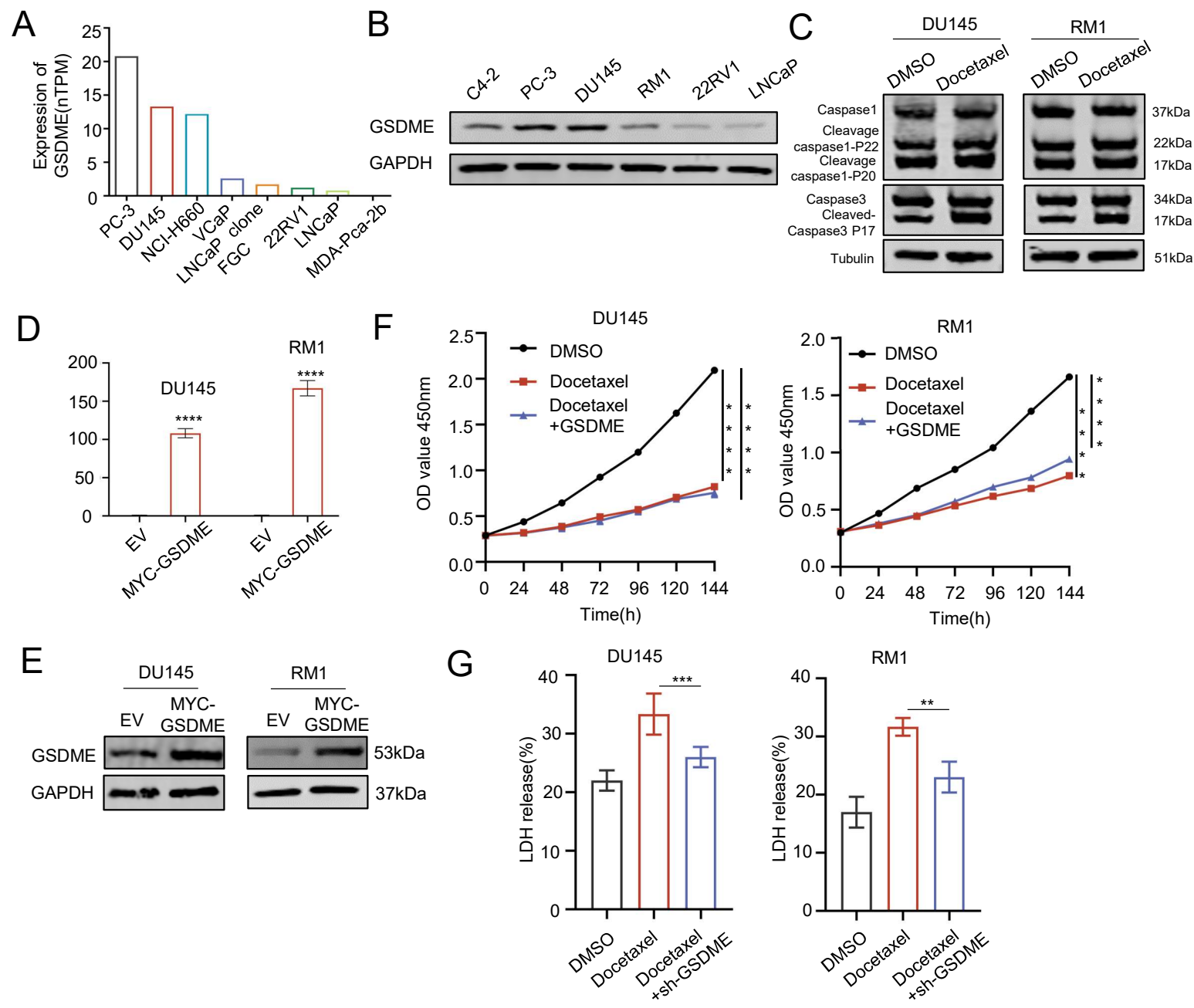

Supplement: Supplementary file 1 — Supplementary Material 1: Supplementary Fig 1. Docetaxel affects immune lymphocytes changes. A: Proportion of immune lymphocytes during non-small cell lung cancer and breast cancer chemotherapy cycle. B: The overlapping Venn diagrams of immune-related genes and docetaxel influencing genes. C: Relationship between related genes and immune cells in prostate tumors. n.s., no significance; *P < 0.05, ***P < 0.001,****P < 0.0001. Supplementary Fig 2. Docetaxel can induce a shift in the mode of death of prostate cancer cells. A: Schematic diagram of a mouse subcutaneous tumor model. B: Survival curves of mice in each group. Data represented as mean ± SD (n = 3). C: Experiment of Cell Plate Cloning under Drug Intervention; D: Quantitative Statistics of Colony Formation in Plate Cloning Experiment. Data represented as mean ± SD (n = 3). n.s., no significance; **P < 0.01, ***P < 0.001, ****P < 0.0001. Supplementary Fig 3. Docetaxel can induce a shift in the mode of death of prostate cancer cells. A: mRNA expression levels of GSDME in prostate cancer cell lines from HPA. B: protein expression levels of GSDME in prostate cancer cell lines. C: Changes of protein expression of Caspase1 and Caspase3 before and after docetaxel treatment D: mRNA expression levels of GSDME after GSDME overexpression. E: protein expression levels of GSDME after GSDME overexpression. F: CCK8 assay on the OD value in 96-well plates of the RM1 cells and DU145 cells after GSDME overexpression. G: Colony formation assay on colony numbers of the RM1 cells and DU145 cells after GSDME overexpression. Data represented as mean ± SD (n = 3). n.s., no significance;**P < 0.01, ***P < 0.001, ****P < 0.0001. Supplementary Fig 4. SKP2 ubiquitinates GSDME and promotes its degradation. A: protein expression levels of GSDME after adding different drugs. B: protein expression levels of GSDME in the condition of docetaxel and TFA. C: protein expression levels of GSDME in the condition of docetaxel and SKP2. D: The degree [file 13046_2025_3614_MOESM1_ESM.zip › Supplementary material/supplement figure3.pdf]

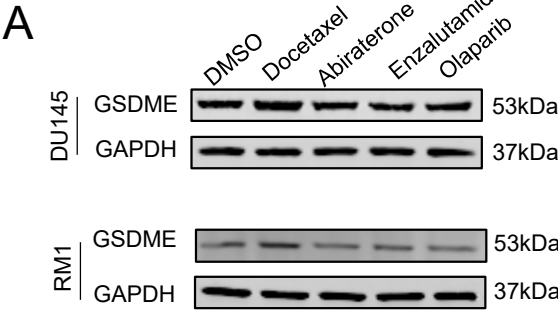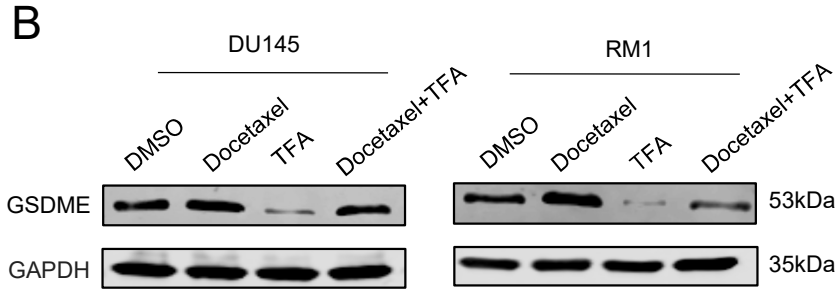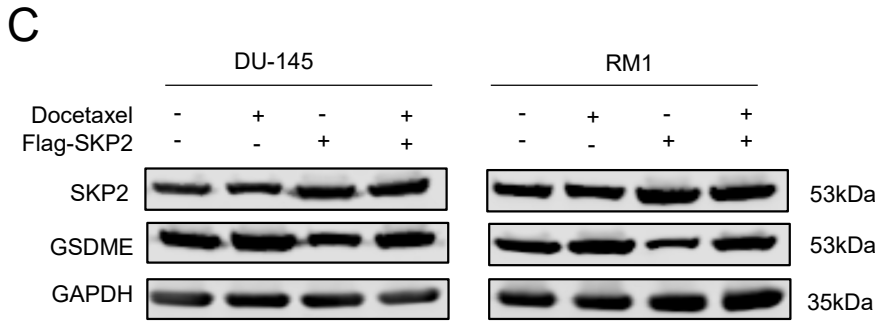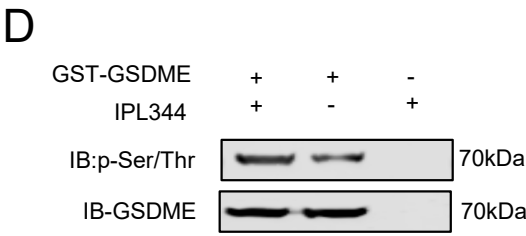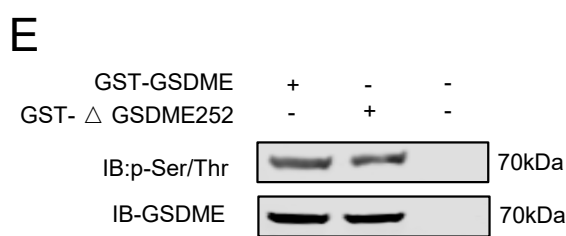

Supplement: Supplementary file 1 — Supplementary Material 1: Supplementary Fig 1. Docetaxel affects immune lymphocytes changes. A: Proportion of immune lymphocytes during non-small cell lung cancer and breast cancer chemotherapy cycle. B: The overlapping Venn diagrams of immune-related genes and docetaxel influencing genes. C: Relationship between related genes and immune cells in prostate tumors. n.s., no significance; *P < 0.05, ***P < 0.001,****P < 0.0001. Supplementary Fig 2. Docetaxel can induce a shift in the mode of death of prostate cancer cells. A: Schematic diagram of a mouse subcutaneous tumor model. B: Survival curves of mice in each group. Data represented as mean ± SD (n = 3). C: Experiment of Cell Plate Cloning under Drug Intervention; D: Quantitative Statistics of Colony Formation in Plate Cloning Experiment. Data represented as mean ± SD (n = 3). n.s., no significance; **P < 0.01, ***P < 0.001, ****P < 0.0001. Supplementary Fig 3. Docetaxel can induce a shift in the mode of death of prostate cancer cells. A: mRNA expression levels of GSDME in prostate cancer cell lines from HPA. B: protein expression levels of GSDME in prostate cancer cell lines. C: Changes of protein expression of Caspase1 and Caspase3 before and after docetaxel treatment D: mRNA expression levels of GSDME after GSDME overexpression. E: protein expression levels of GSDME after GSDME overexpression. F: CCK8 assay on the OD value in 96-well plates of the RM1 cells and DU145 cells after GSDME overexpression. G: Colony formation assay on colony numbers of the RM1 cells and DU145 cells after GSDME overexpression. Data represented as mean ± SD (n = 3). n.s., no significance;**P < 0.01, ***P < 0.001, ****P < 0.0001. Supplementary Fig 4. SKP2 ubiquitinates GSDME and promotes its degradation. A: protein expression levels of GSDME after adding different drugs. B: protein expression levels of GSDME in the condition of docetaxel and TFA. C: protein expression levels of GSDME in the condition of docetaxel and SKP2. D: The degree [file 13046_2025_3614_MOESM1_ESM.zip › Supplementary material/supplement figure4.pdf]

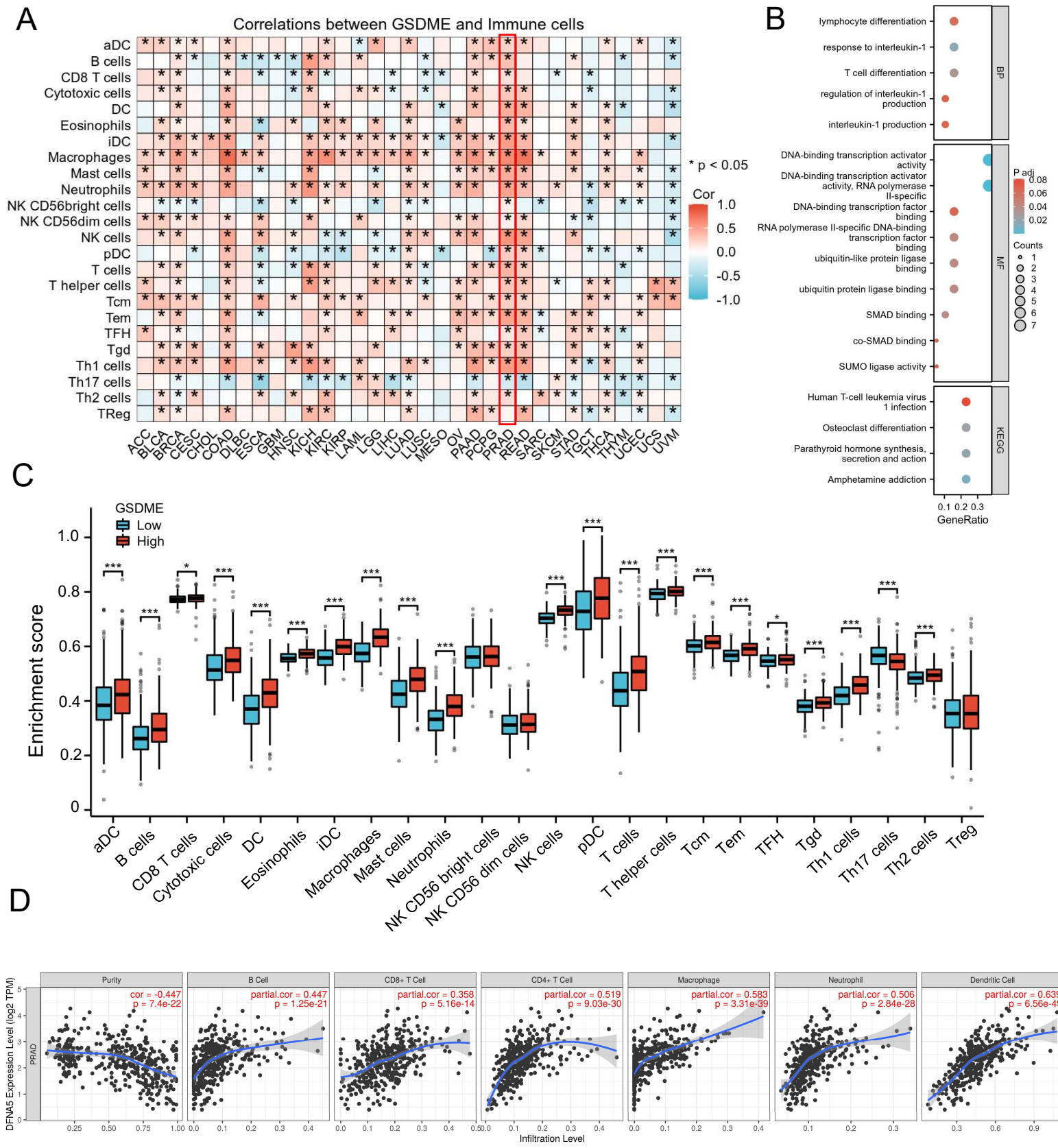

Supplement: Supplementary file 1 — Supplementary Material 1: Supplementary Fig 1. Docetaxel affects immune lymphocytes changes. A: Proportion of immune lymphocytes during non-small cell lung cancer and breast cancer chemotherapy cycle. B: The overlapping Venn diagrams of immune-related genes and docetaxel influencing genes. C: Relationship between related genes and immune cells in prostate tumors. n.s., no significance; *P < 0.05, ***P < 0.001,****P < 0.0001. Supplementary Fig 2. Docetaxel can induce a shift in the mode of death of prostate cancer cells. A: Schematic diagram of a mouse subcutaneous tumor model. B: Survival curves of mice in each group. Data represented as mean ± SD (n = 3). C: Experiment of Cell Plate Cloning under Drug Intervention; D: Quantitative Statistics of Colony Formation in Plate Cloning Experiment. Data represented as mean ± SD (n = 3). n.s., no significance; **P < 0.01, ***P < 0.001, ****P < 0.0001. Supplementary Fig 3. Docetaxel can induce a shift in the mode of death of prostate cancer cells. A: mRNA expression levels of GSDME in prostate cancer cell lines from HPA. B: protein expression levels of GSDME in prostate cancer cell lines. C: Changes of protein expression of Caspase1 and Caspase3 before and after docetaxel treatment D: mRNA expression levels of GSDME after GSDME overexpression. E: protein expression levels of GSDME after GSDME overexpression. F: CCK8 assay on the OD value in 96-well plates of the RM1 cells and DU145 cells after GSDME overexpression. G: Colony formation assay on colony numbers of the RM1 cells and DU145 cells after GSDME overexpression. Data represented as mean ± SD (n = 3). n.s., no significance;**P < 0.01, ***P < 0.001, ****P < 0.0001. Supplementary Fig 4. SKP2 ubiquitinates GSDME and promotes its degradation. A: protein expression levels of GSDME after adding different drugs. B: protein expression levels of GSDME in the condition of docetaxel and TFA. C: protein expression levels of GSDME in the condition of docetaxel and SKP2. D: The degree [file 13046_2025_3614_MOESM1_ESM.zip › Supplementary material/supplement figure5.pdf]

A

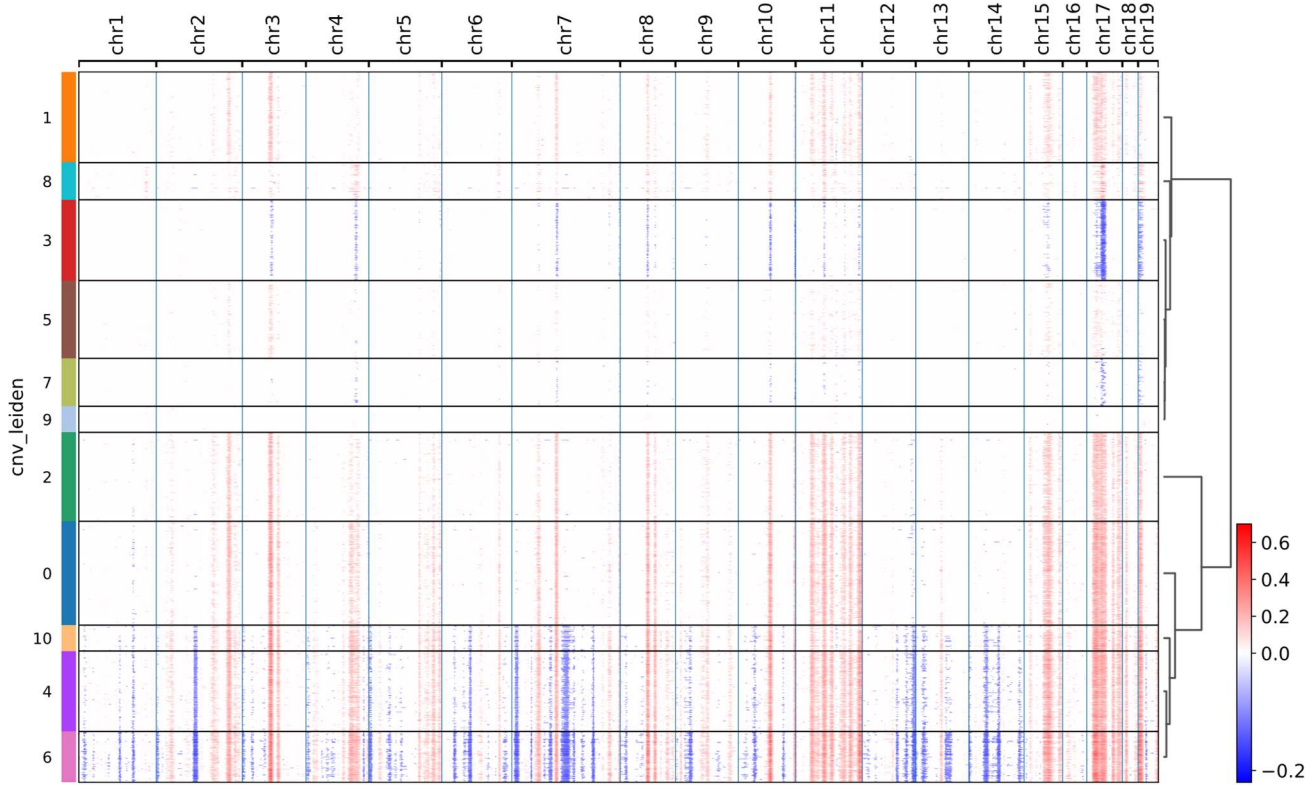

B

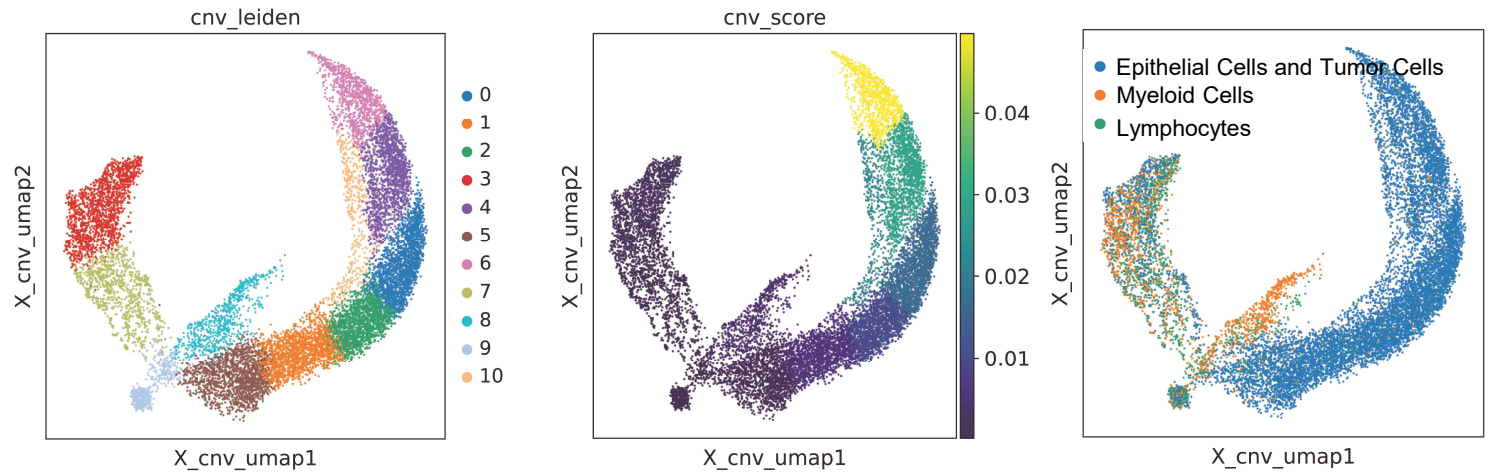

C

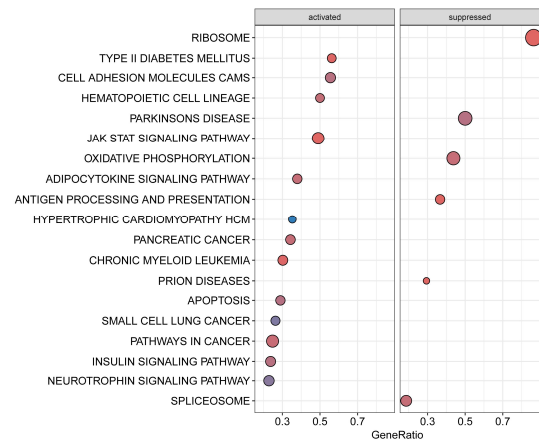

D

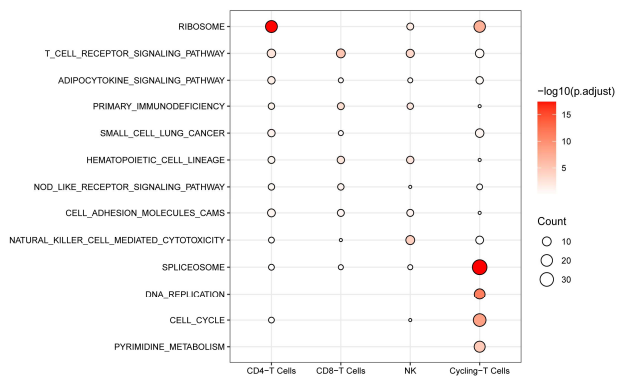

E

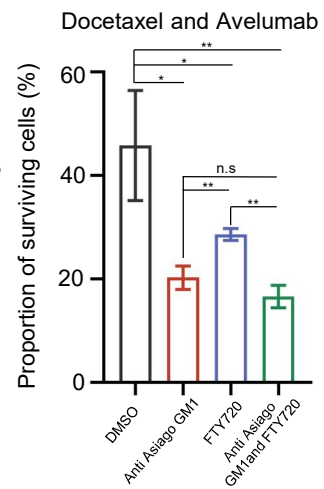

Supplement: Supplementary file 1 — Supplementary Material 1: Supplementary Fig 1. Docetaxel affects immune lymphocytes changes. A: Proportion of immune lymphocytes during non-small cell lung cancer and breast cancer chemotherapy cycle. B: The overlapping Venn diagrams of immune-related genes and docetaxel influencing genes. C: Relationship between related genes and immune cells in prostate tumors. n.s., no significance; *P < 0.05, ***P < 0.001,****P < 0.0001. Supplementary Fig 2. Docetaxel can induce a shift in the mode of death of prostate cancer cells. A: Schematic diagram of a mouse subcutaneous tumor model. B: Survival curves of mice in each group. Data represented as mean ± SD (n = 3). C: Experiment of Cell Plate Cloning under Drug Intervention; D: Quantitative Statistics of Colony Formation in Plate Cloning Experiment. Data represented as mean ± SD (n = 3). n.s., no significance; **P < 0.01, ***P < 0.001, ****P < 0.0001. Supplementary Fig 3. Docetaxel can induce a shift in the mode of death of prostate cancer cells. A: mRNA expression levels of GSDME in prostate cancer cell lines from HPA. B: protein expression levels of GSDME in prostate cancer cell lines. C: Changes of protein expression of Caspase1 and Caspase3 before and after docetaxel treatment D: mRNA expression levels of GSDME after GSDME overexpression. E: protein expression levels of GSDME after GSDME overexpression. F: CCK8 assay on the OD value in 96-well plates of the RM1 cells and DU145 cells after GSDME overexpression. G: Colony formation assay on colony numbers of the RM1 cells and DU145 cells after GSDME overexpression. Data represented as mean ± SD (n = 3). n.s., no significance;**P < 0.01, ***P < 0.001, ****P < 0.0001. Supplementary Fig 4. SKP2 ubiquitinates GSDME and promotes its degradation. A: protein expression levels of GSDME after adding different drugs. B: protein expression levels of GSDME in the condition of docetaxel and TFA. C: protein expression levels of GSDME in the condition of docetaxel and SKP2. D: The degree [file 13046_2025_3614_MOESM1_ESM.zip › Supplementary material/supplement figure6.pdf]
