# Supplementary material for: Docetaxel induced activation of GSDME pathway and pyroptosis enhance immune lethality in prostate cancer cells
Source: J Exp Clin Cancer Res. 2025 Dec 18;45:22. doi: 10.1186/s13046-025-03614-1 (PMC12825250; doi:10.1186/s13046-025-03614-1)

Fig3B

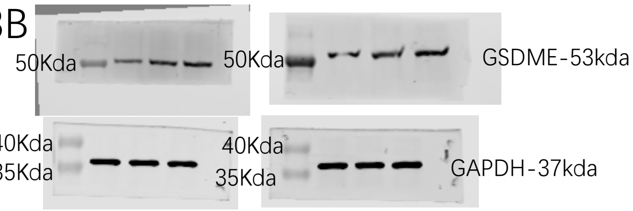

Fig3C

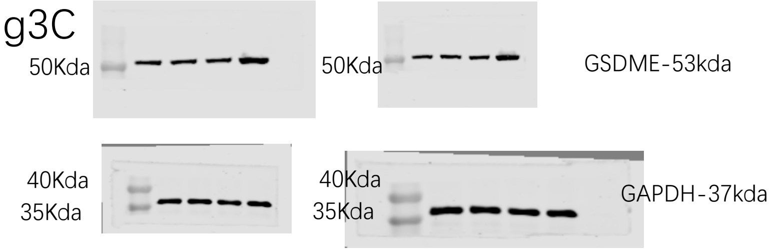

Fig3D

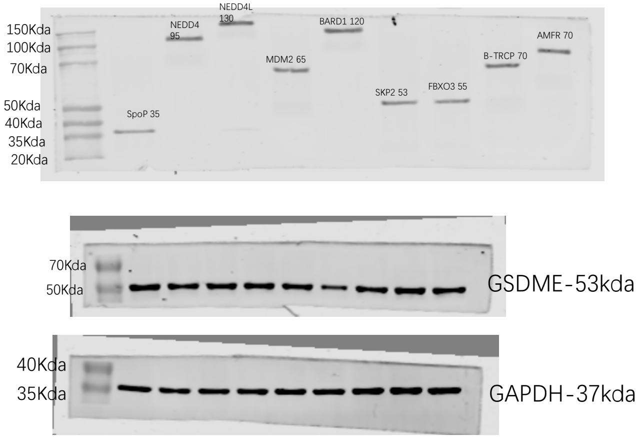

Fig3E

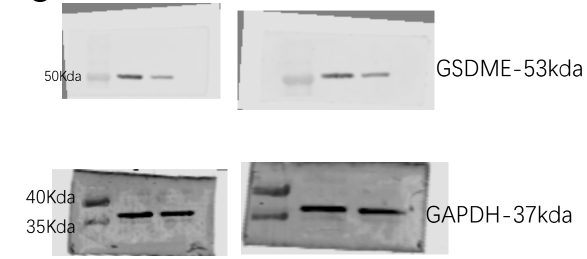

Fig3F

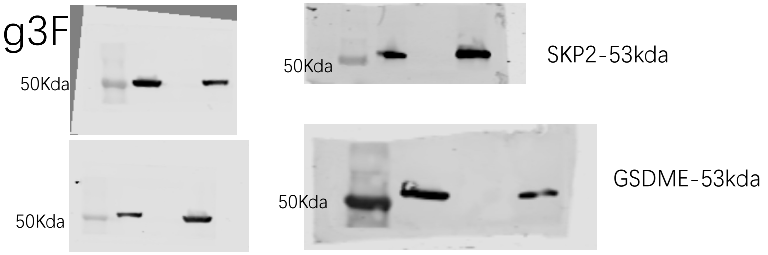

Fig3G

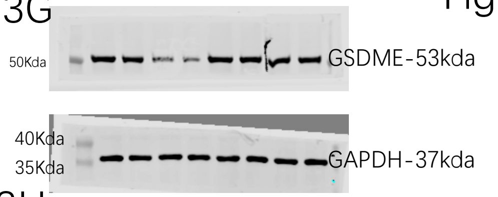

Fig3H

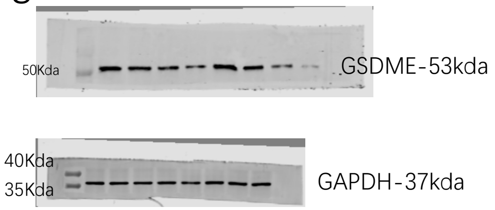

Fig3J

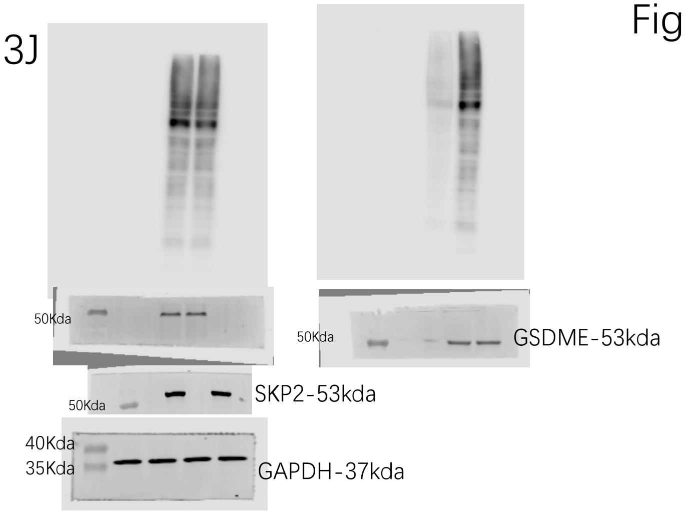

Fig3K

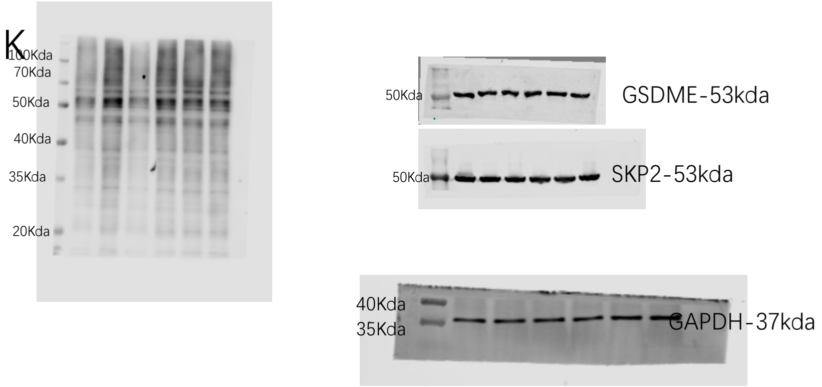

Fig4C

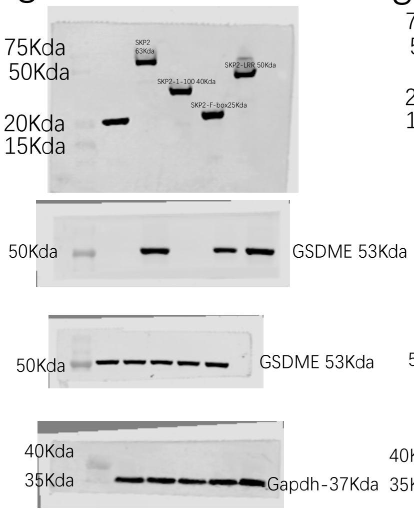

Fig4D

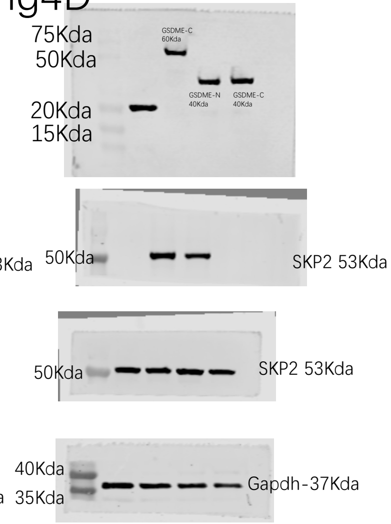

Fig4I

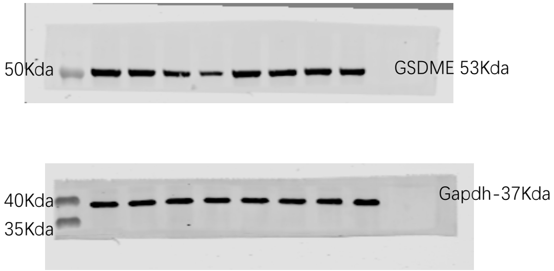

Fig4G

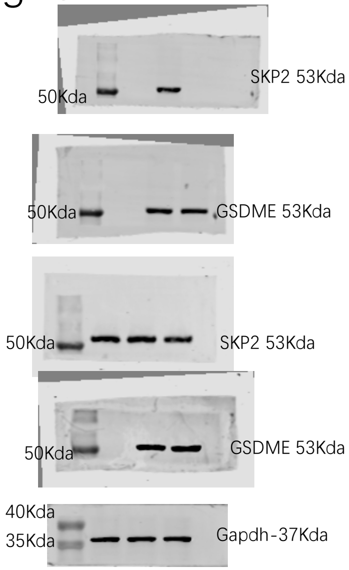

Fig4H

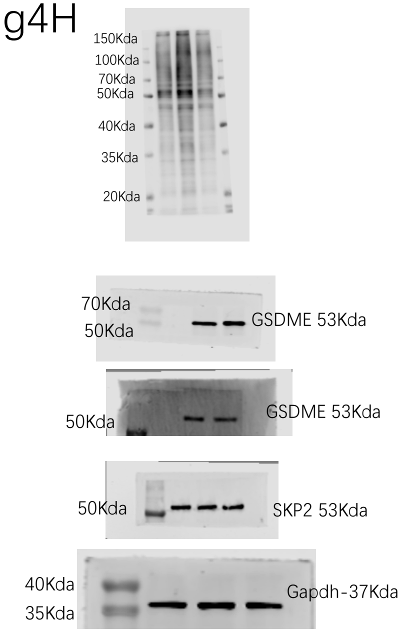

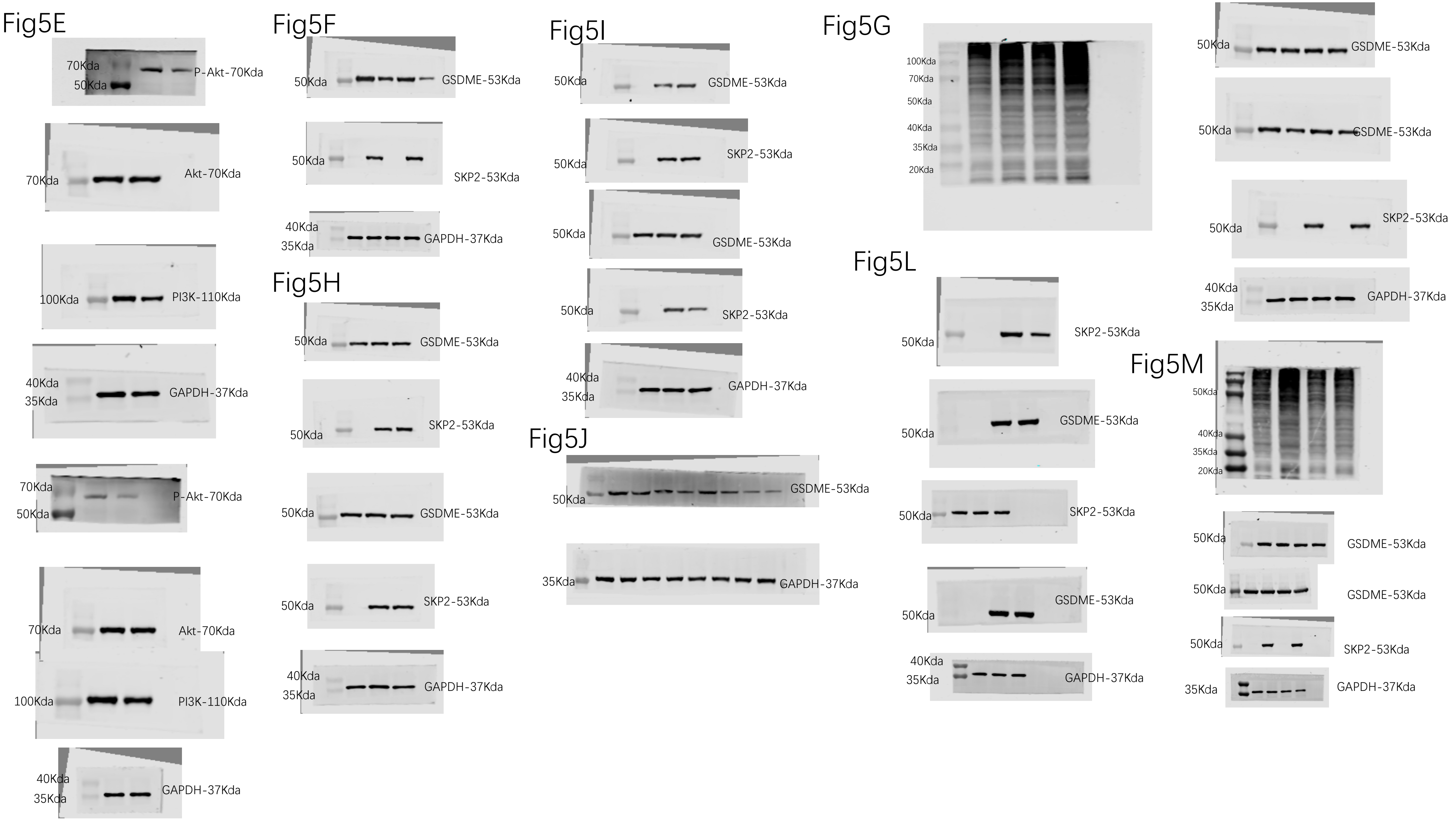
Fig5E
70Kda
50Kda
P-Akt-70Kda
70Kda
Akt-70Kda
100Kda
PI3K-110Kda
40Kda
35Kda
GAPDH-37Kda
70Kda
50Kda
P-Akt-70Kda
70Kda
Akt-70Kda
100Kda
PI3K-110Kda
40Kda
35Kda
GAPDH-37Kda
Fig5F
50Kda
GSDME-53Kda
50Kda
SKP2-53Kda
40Kda
35Kda
GAPDH-37Kda
Fig5H
50Kda
GSDME-53Kda
50Kda
SKP2-53Kda
50Kda
GSDME-53Kda
50Kda
SKP2-53Kda
40Kda
35Kda
GAPDH-37Kda
Fig5I
50Kda
GSDME-53Kda
50Kda
SKP2-53Kda
50Kda
GSDME-53Kda
50Kda
SKP2-53Kda
40Kda
35Kda
GAPDH-37Kda
Fig5J
50Kda
GSDME-53Kda
35Kda
GAPDH-37Kda
Fig5G
100Kda
70Kda
50Kda
40Kda
35Kda
20Kda
50Kda
GSDME-53Kda
50Kda
GSDME-53Kda
50Kda
SKP2-53Kda
40Kda
35Kda
GAPDH-37Kda
Fig5L
50Kda
SKP2-53Kda
50Kda
GSDME-53Kda
50Kda
SKP2-53Kda
50Kda
GSDME-53Kda
40Kda
35Kda
GAPDH-37Kda
Fig5M
50Kda
40Kda
35Kda
20Kda
50Kda
GSDME-53Kda
50Kda
GSDME-53Kda
50Kda
SKP2-53Kda
35Kda
GAPDH-37Kda

Supplement Figure3B

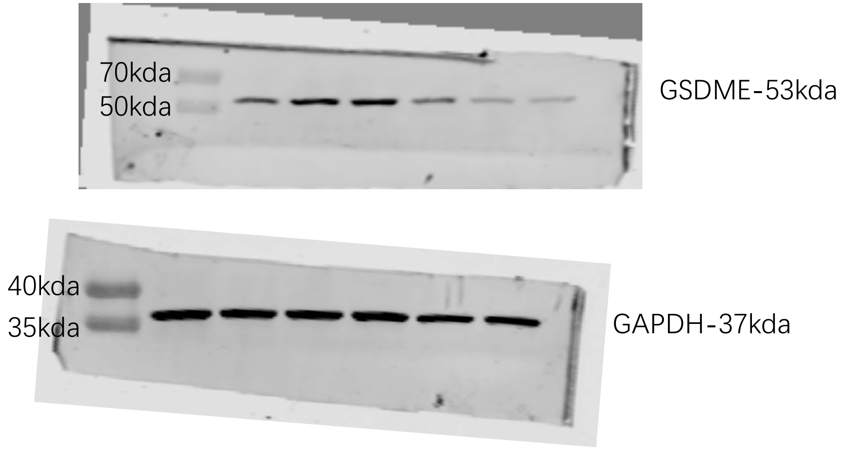

Supplement Figure3C

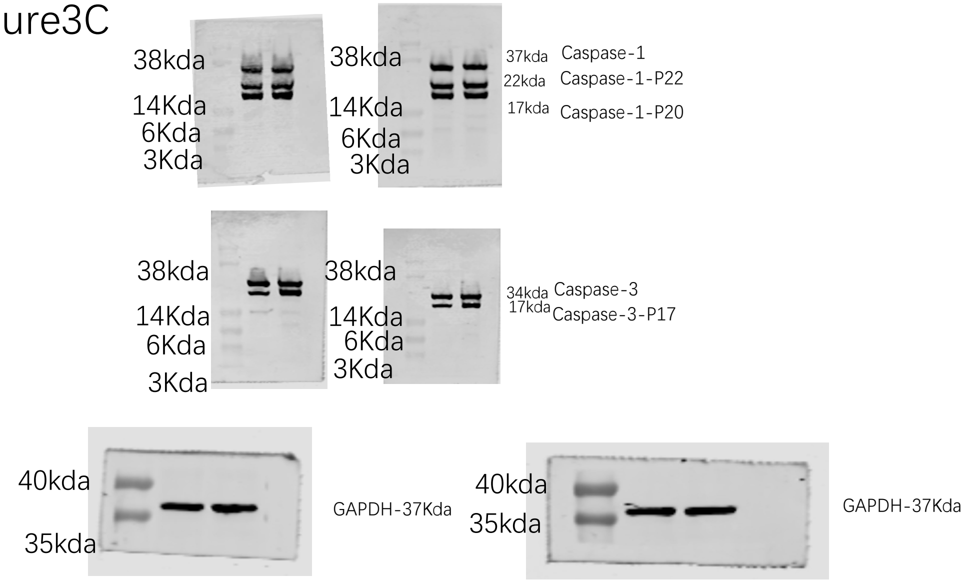

Supplement Figure3E

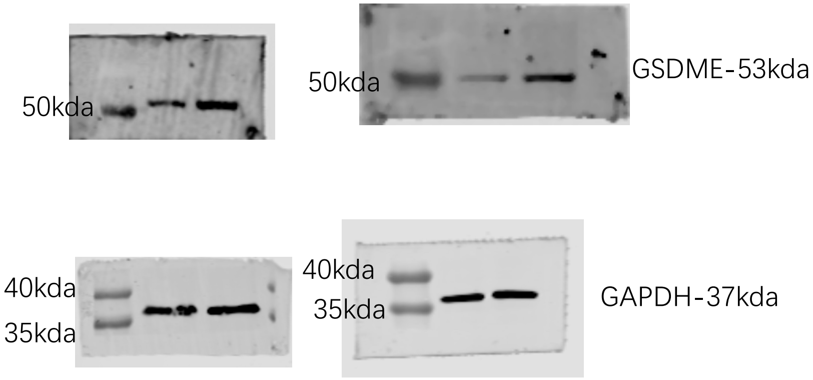

Supplement Figure4A

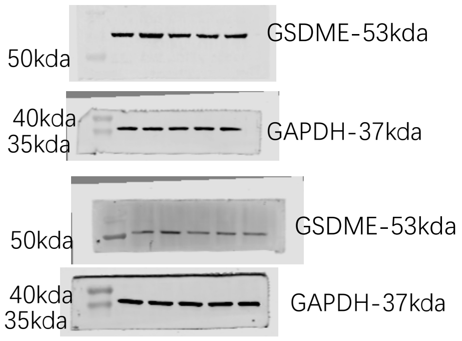

Supplement Figure4B

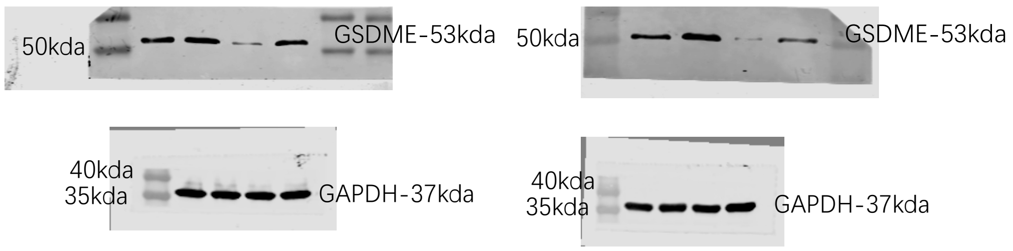

Supplement Figure4C

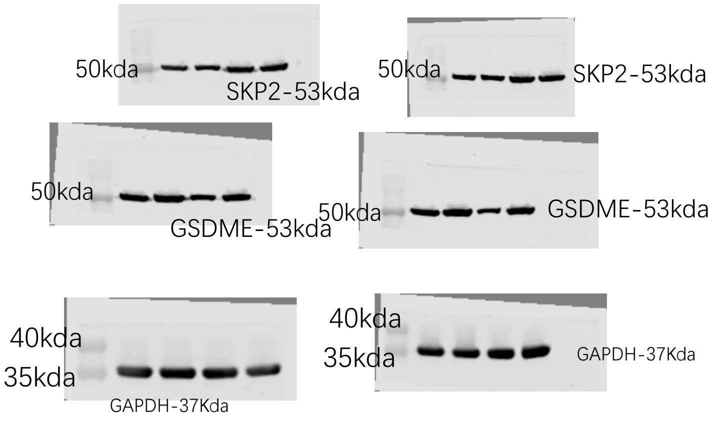

Supplement Figure4E

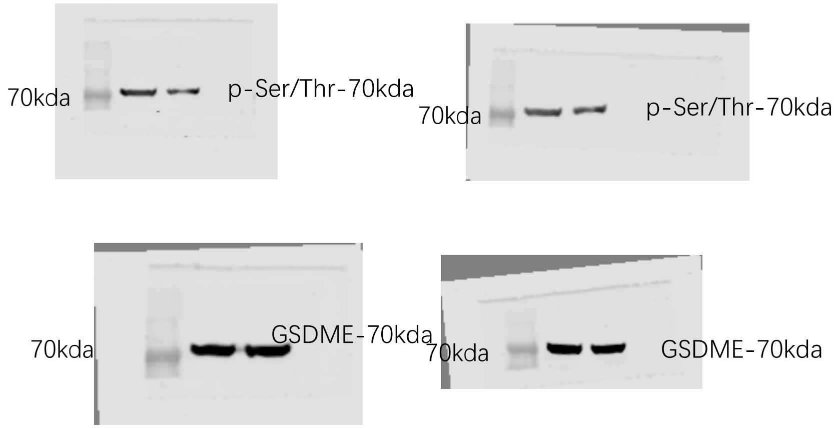

Supplement: Supplementary file 2 — Supplementary Material 2. [file 13046_2025_3614_MOESM2_ESM.pdf]
